# UPLC-qTOF-MS/MS profiling of phenolic compounds in *Fagonia arabica* L. and evaluation of their cholinesterase inhibition potential through in-vitro and in-silico approaches

**DOI:** 10.1038/s41598-025-86227-0

**Published:** 2025-02-12

**Authors:** Sarah A. Badawy, Ahmed R. Hassan, Marwa S. Abu Bakr, Abd El-Salam I. Mohammed

**Affiliations:** 1 Medicinal and Aromatic Plants Department, Desert Research Center, El-Matariya 11753, Cairo, Egypt; 2 Department of Pharmacognosy, Faculty of Pharmacy (for Girls), Al-Azhar University, Nasr City, Cairo, 11651 Egypt; 3 Department of Pharmacognosy, Faculty of Pharmacy (for Boys), Al-Azhar University, Nasr City, Cairo, 13129 Egypt

**Keywords:** *Fagonia Arabica*, UPLC-qTOF-MS/MS, Flavonoids, Butyrylcholinesterase inhibitors, Acetylcholinesterase inhibitors, Docking study, Computational chemistry, Mass spectrometry

## Abstract

*Fagonia arabica* L. is a widely used traditional medicinal herb. This study explored the flavonoid and phenolic acid content in the aerial parts of *F. arabica*, leading to the tentative identification of 42 compounds using Ultra-Performance Liquid Chromatography-Quadrupole Time-of-Flight Mass/Mass Spectrometry and analyzed with the phytochemical-focused RIKEN tandem mass spectral database (ReSpect) for identification based on authentic standards. The total phenolic and flavonoid content was measured in the ethyl acetate and butanol fractions. The flavonoid content in the ethyl acetate fraction was 101 ± 1.43 µg Rutin/mg, compared to 6.48 ± 0.29 µg rutin/mg in the butanol fraction. Similarly, the ethyl acetate fraction contained 199.14 ± 1.58 µg gallic acid/mg, while the butanol fraction had 47.69 ± 0.54 µg gallic acid/mg. Also, the study demonstrated the effectiveness of the different fractions of *Fagonia arabica* L. in inhibiting the butyrylcholinesterase enzyme, which is a key contributor to the progression of Alzheimer’s disease. At a concentration of 0.45 mg/mL, the ethyl acetate fraction showed the highest efficiency, inhibiting butyrylcholinesterase by 50% (IC_50_). Based on the in vitro results, a molecular docking study suggested the selectivity of the tentatively identified compounds towards butyrylcholinesterase over acetylcholinesterase, as kaempferol-3-*O*-glucoside achieved the highest selectivity. This insight could inform potential modifications to enhance selectivity, which may be applied in the synthesis, semi-synthesis, and development of novel treatments for Alzheimer’s disease.

**Supplementary Information:**

The online version contains supplementary material available at 10.1038/s41598-025-86227-0.

## Introduction

The most prevalent type of dementia, Alzheimer’s disease (AD), is a complex and multifaceted age-related neurodegenerative illness that frequently results in significant behavioural symptoms like irritability, anxiety, and sadness in addition to cognitive loss. Numerous possible medications that target the various hypotheses that have been proposed for the treatment of AD have been tried. These hypotheses include cholinergic, amyloid, tau, neuroinflammation, and oxidative stress hypotheses. The cholinergic hypothesis, which contends that AD starts as a shortage in the neurotransmitter acetylcholine synthesis, is the oldest and primary basis for the majority of currently accessible pharmacological therapy. As a result, the use of acetylcholinesterase (AChE) and butyrylcholinesterase (BChE) inhibitors in managing AD has increased significantly^1,2^.

There are structural similarities between the two cholinergic enzymes, including the existence of a catalytic core. Finding novel cholinesterase inhibitors seems to be an essential approach in the fight against AD and related dementias. For this reason, AChE inhibitors like galantamine, donepezil, rivastigmine, and huperzine A continue to be the preferred option for treating AD. Donepezil is a noncompetitive AChE inhibitor that is reversible, blood-brain barrier permeable, and registered for the treatment of all complications levels of AD. Despite that significant side effects such as nausea, vomiting, and bradycardia have limited its clinical benefits. Furthermore, a higher dose increases the frequency and intensity of side effects. As the condition worsens, the low tolerance makes it harder to maintain its effectiveness and necessitates ongoing dose titration. Its greatest therapeutic applicability for AD patients has been severely restricted due to its low safety margin^3^. However, the activity levels of AChE can drop by 85% as AD progresses, and the BChE to AChE ratio can drastically alter between 1:5 and 11:1. The observed activity inversion with these two proteins suggests that the more the selective inhibition of BChE, the more levels of acetylcholine in the impaired brain. This is because the sequestering effect of AChE to the drug due to its non-specific binding which may far the drug away from its intended target, with minimal improvement in the reduced levels of acetylcholine. Furthermore, even while AChE may no longer be active in the brain, it is still there and linked to the development of amyloid-beta plaques in AD patients, which may make the medication sequestering problem worse^4–6^.

Plant phenolic constituents such as flavonoids have been reported to be potent AChE inhibitors for treating AD^7^. It was found that eight flavonoids, including kaempferol, galangin, quercetin, myricetin, apigenin, rutin, fisetin, and luteolin, can reversibly inhibit human BChE^8^. Furthermore, their efficacy was explained by some hydrophobic interactions between these ligands and AChE and BChE^9^. It has been reported that *Fagonia arabica* L. is a natural source of phenolic compounds as two flavonoid glycosides: acacetin-7-*O*-rhamnoside and kaempferol-7-*O*-rhamnoside, were recovered from the ethyl acetate fraction of *F. arabica*^10^. Herbacetin-8-methyl ether-3-*O*-rutinoside, herbacetin-8-rutinoside, herbacetin-3,7-*O*-diglucoside, herbacetin-3-*O*-rutinoside-7-*O*-glucoside, isorhamnetin-3-*O*-rutinoside and isorhamnetin-3-*O*-glucoside were also reported^11^. *F. arabica* L. is a species of the genus *Fagonia* (Zygophyllaceae), which includes wild flowering plants found in India, the Middle East, Africa, and the Mediterranean Basin. *F. arabica*is a tiny underbrush with prickly spines that has stiff, slightly prostrate branches. It has small, pink flowers, whole, linear-elliptic leaflets, and one to three foliolates grouped opposite one other on its opposite leaf stalk^12^. *F. arabica* has reportedly been used as a folk treatment for several illnesses since ancient times. *F. arabica*was also described in Ayurvedic medicine as a blood purifier and deobstruent. In Arabic, it’s called Shawka al-Baidaa, in German, Fagonie, and English, Virgin’s Mantle. Secondary metabolites including glycosides, flavonoids, triterpenes, saponins, and steroids are abundant in it^10,13^.

Metabolite-finger print fragment ions for structural elucidation by MS/MS analysis enable the detection of the metabolic profile of the plant. Therefore, for metabolites, especially phenolic compounds, identification in metabolomics, the creation of an MS/MS data resource and database is necessary. Web resources like the RIKEN tandem mass spectral database (ReSpect for phytochemicals) have recently made the data of MS/MS public. The ReSpect database contains 3595 metabolites, of which 163 literature studies provided 76% of the metabolites. Authentic standards provided the remaining metabolites^14^.

Based on the prior research, the main objective was to investigate the *F. arabica* aerial parts’ specific butyrylcholinesterase inhibitory activity by in-vitro and in-silico experiments. Furthermore, the advanced UPLC-qTOF-MS/MS technology tentatively identified its content from the phenolic compounds responsible for this inhibition activity.

## Experimental

### Chemistry

#### Chemicals and instruments

The following high-purity chemical solvents were purchased: water (Milli-Q) from Millipore, USA; ammonium formate, sodium hydroxide, formic acid, ethyl acetate, *n*-butanol, ethyl acetate, and *n*-hexane, standard rutin and gallic acid from Sigma-Aldrich, Germany, and Fisher Scientific, US. A microplate reader FluoStar Omega was used for measuring the total phenolic and flavonoid content.

#### Plant material

*Fagonia arabica*L. aerial parts (Family: Zygophyllaceae) were gathered in March 2023, Eastern Desert of Egypt at the flowering stage (GPS coordinates: 25°55′–26°00′N, 32°50′–33°00′ E)^15^. The plant specimen was verified by Prof. Dr. Abd El-Halim Abd El-Mogali Mohamed, Flora and Phytotaxonomy Researches Department, Agricultural Museum, Dokki, Giza, Egypt and a voucher specimen of the plant (CAIM-29-3-2023/1) was preserved there in Agricultural Museum’s herbarium, Egypt. Experimental and plant collection protocol was achieved after permission from the “Desert Research Center, Cairo, Egypt” (serial number of the protocol: MP 1841) and all processes comply with relevant institutional, national, and international guidelines and legislation.

#### Extraction and fractionation by liquid-liquid partition chromatography

50 g of *F. arabica* aerial parts were macerated in 80% methanol until complete extraction was achieved. The hydro-methanolic extract was filtered and then concentrated under reduced pressure at 40 °C using a rotary evaporator (rotavapor). The concentrated extract (7 g, 14% of the dry plant material weight) was dissolved in the minimum amount of distilled water. The extract was then sequentially fractionated by liquid-liquid partition chromatography with ethyl acetate (EtOAc) and *n*-butanol (*n*-BuOH) and the rest is the remaining aqueous fraction. All fractions were filtered and concentrated under reduced pressure until dryness yielding 1.98 g EtOAc fraction (28.3% of the 7 g extract), 1.53 g *n*-BuOH fraction (21.86%) and 3.46 g the remaining aqueous fraction (49.43% of the total extract)^16^.

#### Quantitative estimation of total phenolic and flavonoid content

**Fig. 1:**
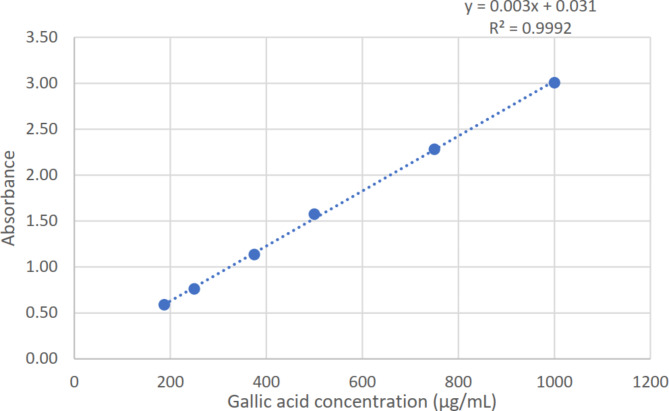
Gallic acid calibration curve for quantitive estimation of total phenolic content.

For analysis, the *n*-butanol and ethyl acetate fractions were produced in methanol at a concentration of 5 mg/mL. The Folin–Ciocalteu technique was used to determine the total phenolic content^17^. In a 96-well microplate, 10 µL of each sample or standard was combined with 100 µL of diluted 1:10 Folin-Ciocalteu reagent for the experiment. Next, 80 µL of 1 M sodium carbonate (Na_2_CO_3_) was added. For 20 min, the mixture was incubated in the dark at room temperature (25 °C). Following incubation, the blue complex’s absorbance at 630 nm was determined. The phenolic concentration of the samples was measured using the gallic acid calibration curve (Fig. 1).

**Fig. 2:**
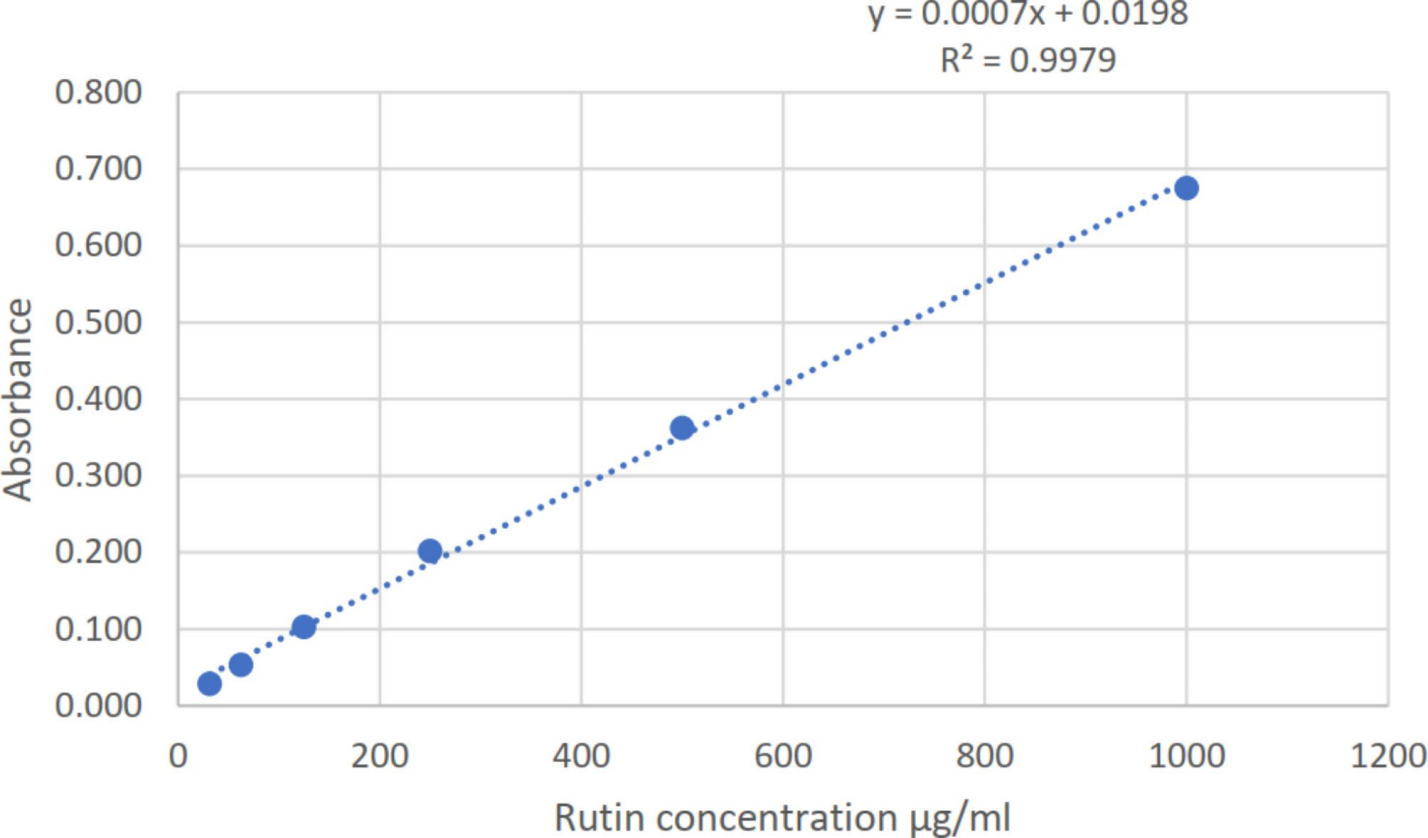
Rutin calibration curve for quantitive estimation of total flavonoid content.

The aluminum chloride technique was used to determine the total flavonoid content^18,19^. To summarize, a 96-well microplate was filled with 15 µL of each sample or standard, 175 µL of methanol, and 30 µL of 1.25% AlCl_3_ solution. The mixture was then incubated for five minutes after 30 µL of 0.125 M sodium acetate (C_2_H_3_NaO_2_) was added. Following incubation, the yellow color’s absorbance was measured at 420 nm. The flavonoid concentration of the samples was measured using the rutin calibration curve (Fig. 2). The results represent the average of three replicate measurements, and all data are reported as means ± standard deviation (SD).

#### LC-qTOF-MS/MS analysis of *F. Arabica* total hydro-methanolic extract

##### Sample preparation

A reconstitution solvent was made out of H_2_O: MeOH: MeCN (acetonitrile), 50:25:25 v/v. 50 milligrams of the dried hydro-methanolic extract was dissolved in one millilitre of the reconstitution solvent. After two minutes of vortexing, ten minutes of ultrasonication, and ten minutes of centrifuging at 10,000 rpm, complete solubility was achieved. Reconstitution solvent was used to dilute the stock solution from 50 to 1000 µL. Ultimately, 2.5 µg/µL was the injected concentration. A 10 µL sample of injection was used for both positive and negative modes. Moreover, inject a blank sample of 10 µL reconstitution solvent.

##### Acquisition method

Solution (A): 5 mM ammonium formate in 1% methanol, pH adjusted with formic acid to 3.0, was the positive mode mobile phase. Solution (B) for the negative mode was made up of 1% methanol and 5 mM ammonium formate, which had been pH-adjusted with sodium hydroxide. Solution (C): For both the negative and positive modes, 100% acetonitrile was utilized. With a flow rate of 0.3 mL/min, the gradient elution was carried out using the following programs: (1) for positive mode: 0–20 min, 95% A and 5% C; 21–28 min, 5% A and 95% C; and 28.1–35 min, 95% A and 5% C, (2) for negative mode: 0–20 min, 95% B and 5% C; 21–28 min, 5% B and 95% C; and 28.1–35 min, 95% B and 5% C. Compounds were separated using an X select HSS T3 (2.5 μm, 2.1 × 150 mm) column (Waters, USA) and in-line filter disks pre-column (0.5 μm x 3.0 mm, Phenomenex, USA) conditioned at 40 °C. Exion LC™ Series UHPLC hardware for chromatographic separation and the information-dependent acquisition (IDA) acquisition was performed on a Triple TOF 5600+ (Sciex, US). LC-Triple TOF control was achieved by Analyst-TF 1.7.1^20^.

##### LC-MS data processing

Utilizing the open-source MS-DIAL 4.9 tool, the material was comprehensively analyzed utilizing small molecules and non-targeting techniques. ReSpect databases were used as reference databases; they contain 2737 records for positive ion mode and 1573 records for negative ion mode^14^. This time, the MasterView 1.1 package in conjunction with the PeakView 2.2 program (AB SCIEX, US) was utilized to validate features (peaks) from the Total Ion Chromatogram (TIC) using the MS-DIAL output. The standards were: sample-to-blank intensities were to be higher than 3, and features had to be aligned with a Signal-to-Noise ratio more than 10 (non-targeted analysis).

### Evaluation of butyrylcholinesterase inhibition activity

#### Chemicals and sample preparation

The butyrylcholinesterase enzyme which was obtained from equine serum [CAT number: C7512], the indicator [DTNB Ellman’s reagent] and the substrate [butyrythiolcholine iodide], all were purchased from Sigma-Aldrich. As a reference, donepezil HCl was produced with a final concentration of 0.004 µg/mL and 0.4 µg/mL in methanol. Samples were dissolved in methanol and evaluated also at six concentrations: 1000, 500, 400, 200, 100 and 50 µg/mL.

#### Butyrylcholine esterase inhibitor assay

With a few minor adjustments, the experiment was performed by previously published protocols^21,22^. First, buffer (1) was prepared by adding 100 mM tris buffer and adjusting the pH to 7.5. Additionally, buffer (2) contains 0.1% bovine serum albumin and is a 50 mM tris buffer with a pH of 7.5. Secondly, 10 µL of the indicator solution (0.4 mM in buffer (1)) was applied to a 96-well plate, followed by 20 µL of the enzyme solution (butyrulcholine esterase 0.02 U/mL in buffer (2)). Third, 140 µL of buffer (1) and 20 µL of the sample/standard solution were added. The mixture was allowed to stand for fifteen minutes at 25 °C. Subsequently, a 10 µL amount of substrate (0.4 mM butyrylthiocholine iodide in buffer 1) was applied to each well. In a dark room, the plate was incubated for 20 min at ambient temperature. The color was measured at 412 nm following the incubation time. The means ± SD of the data are displayed. The findings were recorded using a FluoStar Omega microplate reader.

### Molecular docking

To analyze binding affinities and bond interactions between the discovered phenolic compound and Acetylcholinesterase (AChE_PDB:4EY5) and Butyrylcholinesterase (BChE_PDB:4BDS), the docking was established using AutoDock vina modelling software^6^. PubChem and Chem3D Ultra 8.0 were the source of our phenolic compounds’ 3D structures. To attain optimal outcomes, Chimera 1.17.3 permits the application of energy minimization and protonation before docking. The results were processed using Discovery Studio 2024.

### Statistical analysis

The results of total phenolic and flavonoid content and BChEI (% inhibition) experiments are shown as mean ± standard deviation of the mean for *n* = 3.

## Results and discussion

### Chemistry

#### Quantitative estimation of total phenolic and flavonoid content

##### Total phenolic content

**Fig. 3:**
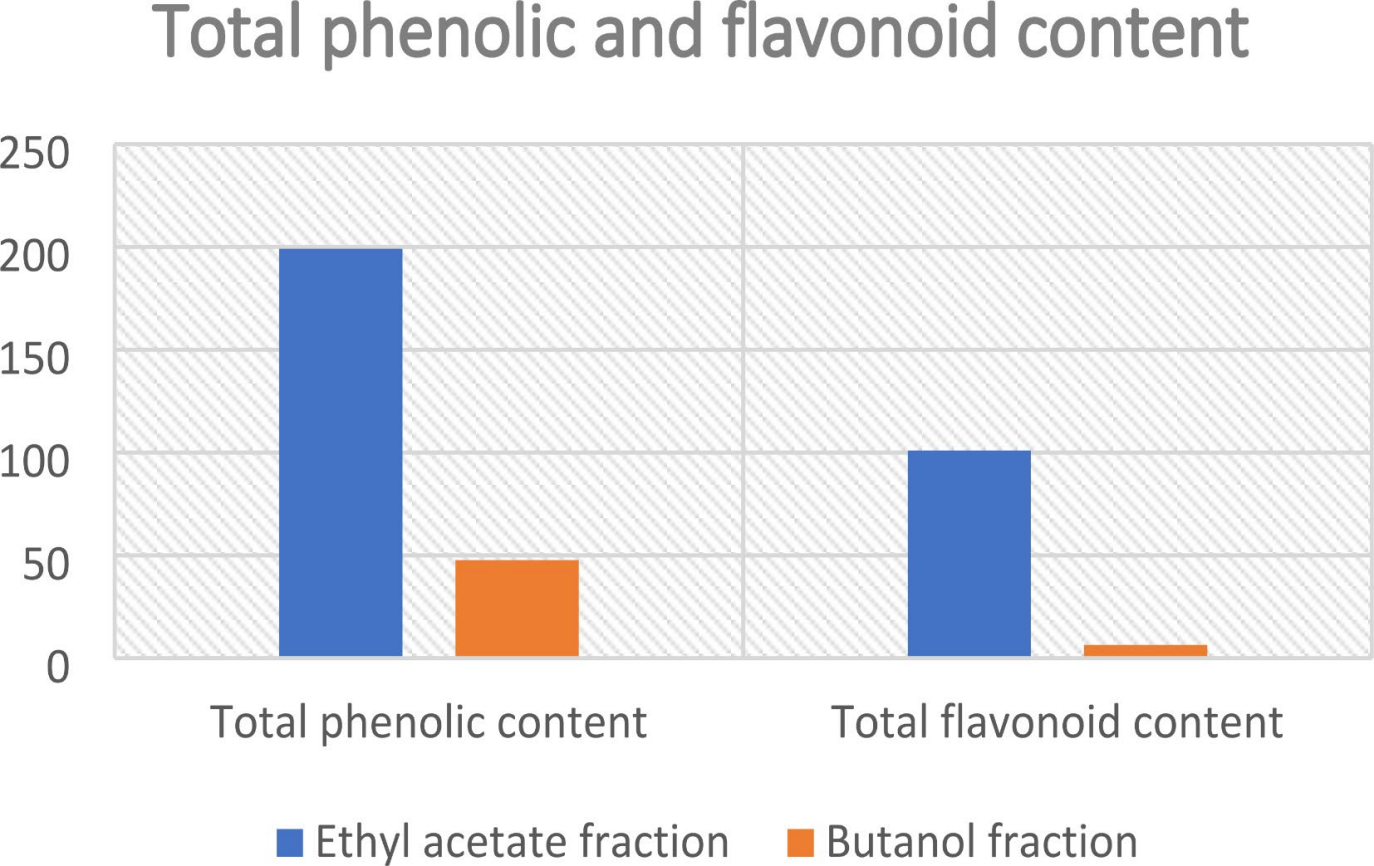
Comparative chart of the total phenolic and flavonoid content of ethyl acetate and butanol fractions.

The total phenolic content was determined using the Folin–Ciocalteu method^17^ as described for ethyl acetate and butanol fractions and the data are represented as means ± SD using gallic acid as a standard. The results showed that ethyl acetate fraction is richer in phenolics than butanol fraction; it contains 199.14 ± 1.58 µg GA/mg Table 1; Fig. 3.

##### Total flavonoid content

The total flavonoid content was determined using the aluminum chloride method as described^18,19^, Data are represented as means ± SD and rutin was used as a standard. Also, the results showed that ethyl acetate fraction is richer in flavonoids than butanol fraction; it contains 101 ± 1.43 µg R/mg Table 1; Fig. 3.

**Table 1:** Results of the total phenolic and flavonoid content of ethyl acetate and butanol fractions.

Fraction	Total phenolic content µg GA/mg sample	Total flavonoid content µg *R*/mg sample
Ethyl acetate	199.14 ± 1.58	101 ± 1.43
Butanol	47.69 ± 0.54	6.48 ± 0.29

As previously understood based on scientific bases, both the ethyl acetate and butanol extracts are abundant in phenolic compounds. To determine which extract exhibits a higher concentration of these compounds, and thus contributes more significantly to the observed biological activity, a comparative analysis was conducted. The results confirmed that the ethyl acetate extract is notably richer in phenolic compounds and therefore demonstrated the greatest inhibitory effect on the butyrylcholinesterase enzyme.

#### UPLCqTOFMS/MS of *F. Arabica* total hydro-methanolic extract

**Fig. 4:**
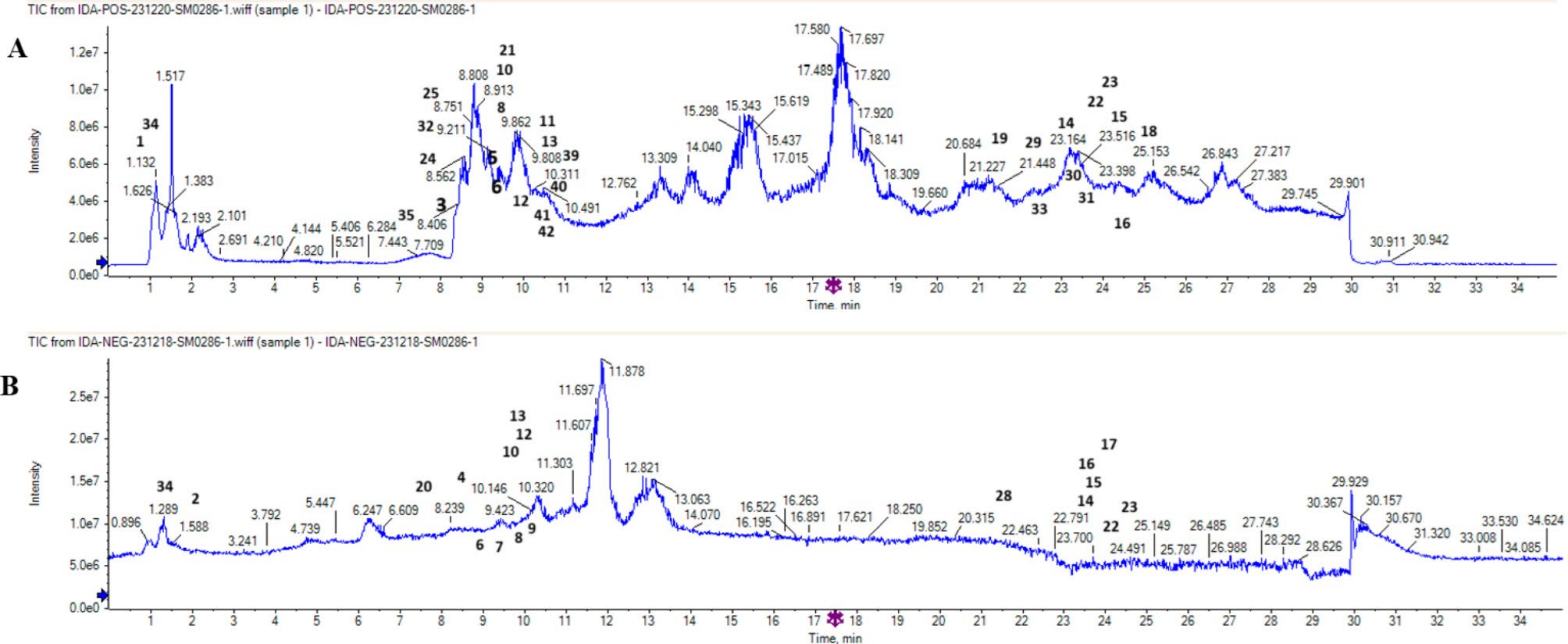
UPLC-qTOF-MS/MS total ion chromatograms (TIC) of *F. arabica* L. aerial parts total hydro-methanolic extract (**A**) total extract in the positive ion mode, (**B**) total extract in the negative ion mode.

**Fig. 5:**
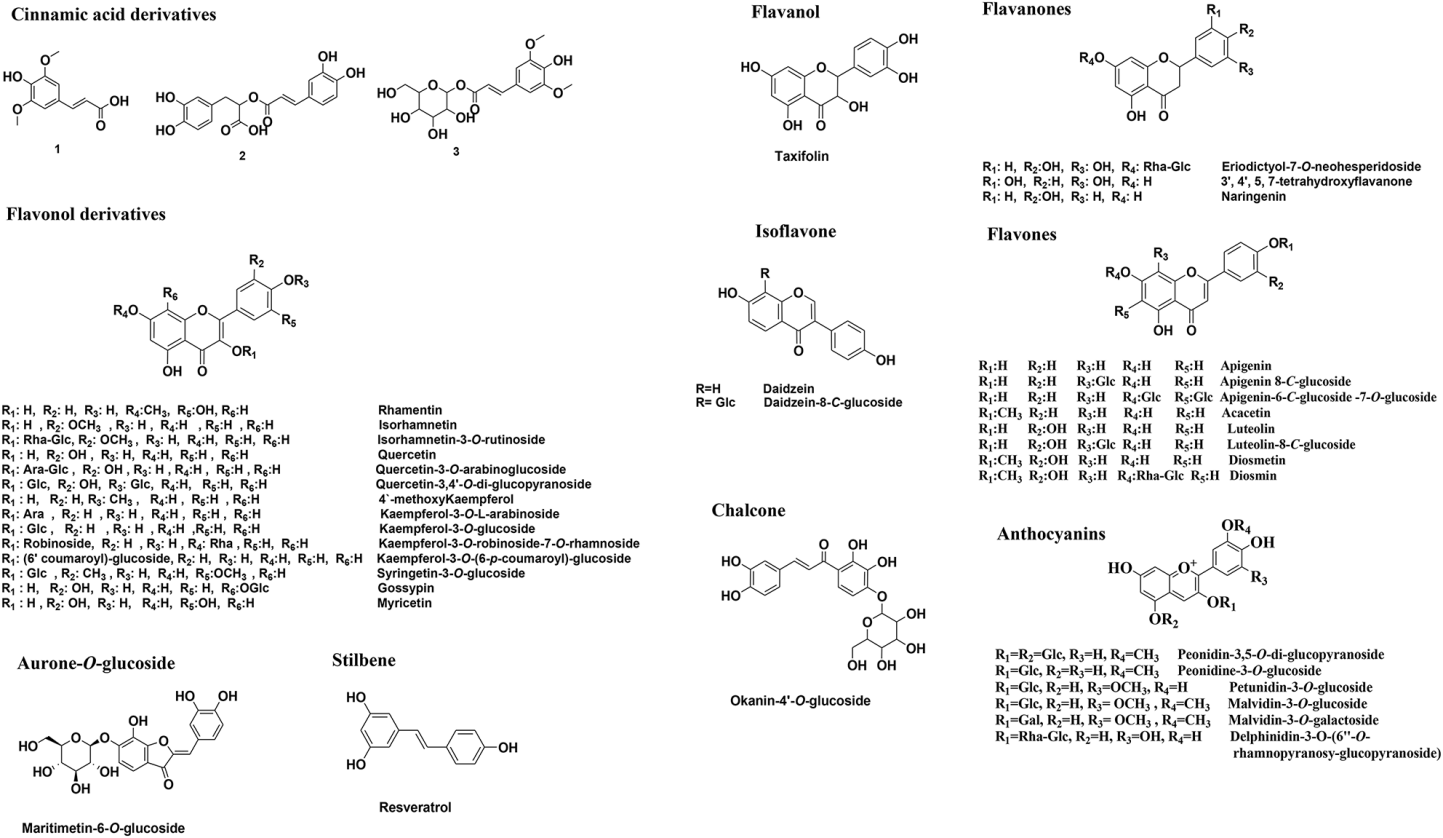
Classification and structures of the major identified peaks using UPLC-qTOF-MS/MS from the total hydro-methanolic extract of *F. arabica* L. aerial parts.

42 phenolic compounds, including 3 phenolic acids (cinnamic acid derivatives), 15 flavonols, 1 flavanol, 4 flavanones, 8 flavones, 2 isoflavones, 1 chalcone, 1 aurone *O*-glycosides, 1 stilbene and 6 anthocyanins (Table 2; Figs. 4 and 5).

##### Phenolic acid derivatives

Phenolic acids and their derivatives are among the most common forms of phytochemical metabolites. These are organic acids with a carboxyl, hydroxyl, or methoxy group immediately attached to an aromatic ring in their chemical structure. Phenolic acids fall into two categories: derivatives of benzoic and cinnamic acids.

The characteristic COO loss (−44 Da) that phenolic acids frequently display can happen in positive and negative ionization modes. Moreover, the fragmentation pattern of phenolic acid glycosides may be explained by the loss of intact sugar residue, resulting in a base peak fragment ion that is associated with the aglycone component.

**Fig. 6:**
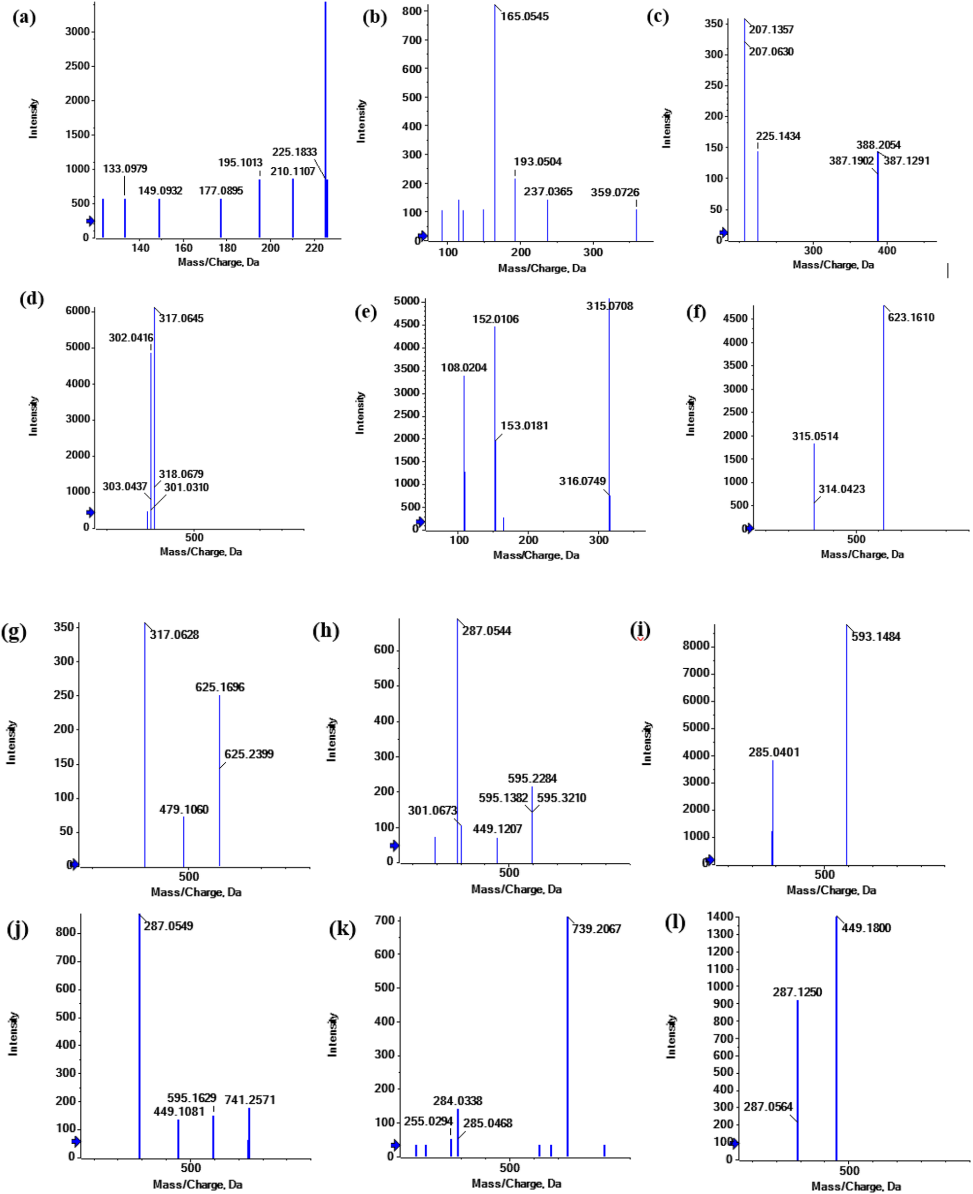

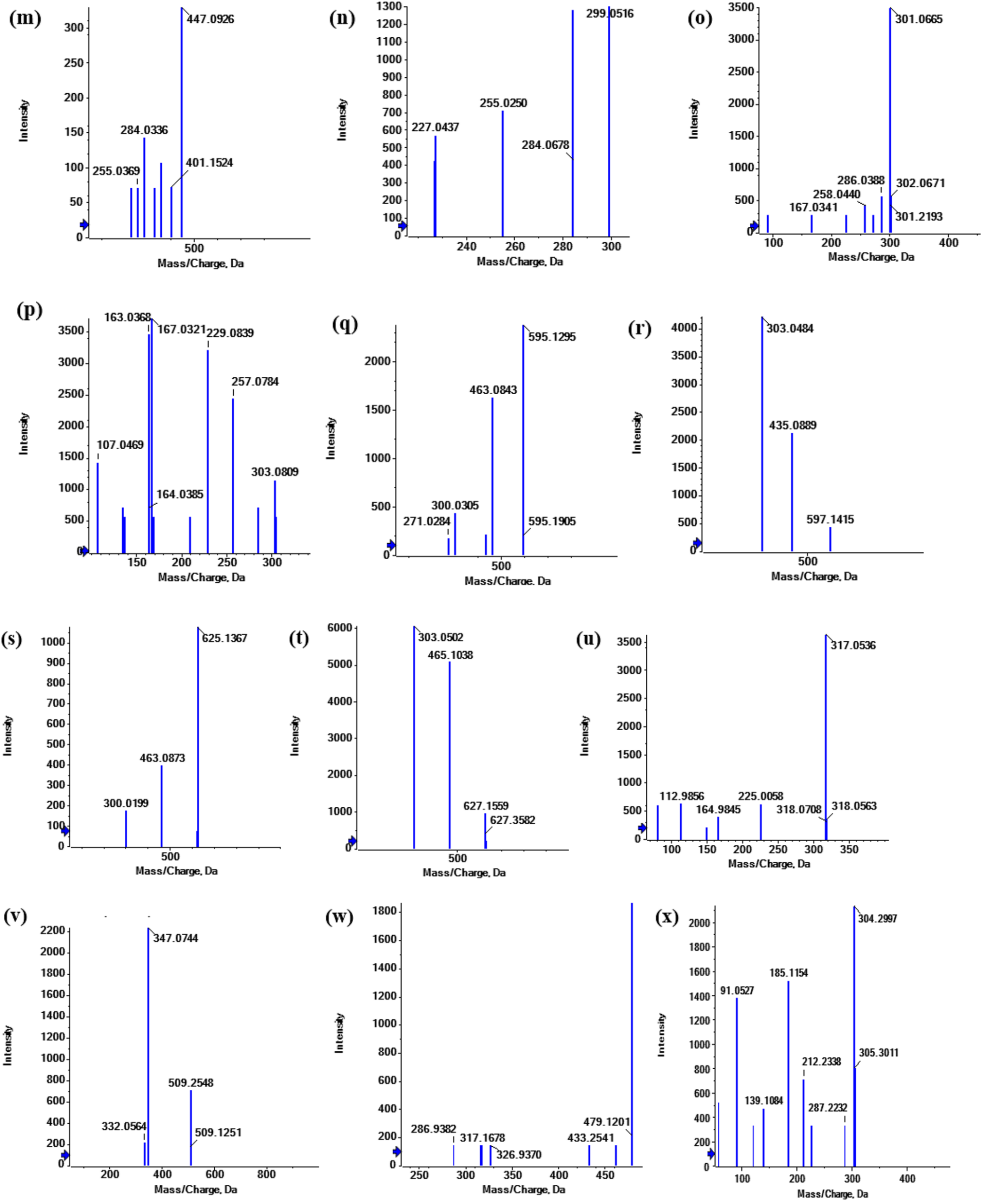
Fragmentation figures of the main identified cinnamic acid derivatives, flavonol and flavanol compounds in *Fagonia arabica* L. hydro-methanolic extract (**a**) MS/MS spectrum of 3-(4-hydroxy-3,5-dimethoxyphenyl)−2-propenoic acid, (**b**) MS/MS spectrum of rosmarinic acid, (**c**) MS/MS spectrum of 1-*O*-sinapoyl-*β*-D-glucose, (**d**), (**e**) MS/MS spectrum of isorhamnetin at both positive and negative mode, respectively, (**f**), (**g**) MS/MS spectrum of isorhamnetin-3-*O*-rutinoside at both negative and positive mode, respectively, (**h**), (**i**) MS/MS spectrum of kaempferol-3-*O*-(6-*p*-coumaroyl)-glucoside at both positive and negative mode, respectively, (**j**), (**k**) MS/MS spectrum of kaempferol-3-*O*-robinoside-7-*O*-rhamnoside at both positive and negative mode, respectively, (**l**), (**m**) MS/MS spectrum of kaempferol-3-*O*-glucoside at both positive and negative mode, respectively, (**n**), (**o**) MS/MS spectrum of 4`-methoxyKaempferol at both negative and positive mode, respectively, (**p**) MS/MS spectrum of quercetin in positive mode, (**q**) MS/MS spectrum of quercetin-3-*O*-arabinoglucoside in negative mode, (**r**) MS/MS spectrum of quercetin-*O*-pentoside-*O*-hexoside in positive mode, (**s**), (**t**) MS/MS spectrum of quercetin-3,4’-*O*-di-*β*-glucopyranoside at both negative and positive mode, respectively, (**u**) MS/MS spectrum of myricetin in negative mode, (**v**) MS/MS spectrum of syringetin-3-*O*-glucoside in positive mode, (**w**) MS/MS spectrum of gossypin in negative mode, (**x**) MS/MS spectrum of (±)taxifolin in positive mode.

As cinnamic acid derivatives, three peaks (1), (2), and (3) were categorized. They were tentatively recognized as 3-(4-hydroxy-3,5-dimethoxyphenyl)−2-propenoic acid, rosmarinic acid and 1-*O*-sinapoyl-*β*-D-glucose (Table 2; Figs. 5 and 6).

**Peak (1)** (Fig. 6a) [Rt 1.05 min, [M + H]^+^ at m/z 225.1833 (C_11_H_13_O_5_)^+^] showed a peak at *m/z* 210 [M + H-15]^+^ which represents the loss of methyl group (-CH_3_) and *m/z* 195 [M + H-2CH_3_]^+^, revealing the natural loss of another methyl residue. It also showed a fragment at *m/z* 177 [M + H-2CH_3_-H_2_O]^+^, representing the additional loss of H_2_O and other fragments at 149 [M + H-2CH_3_-H_2_O-CO]^+^, 133 [M + H-2CH_3_-H_2_O-CO_2_]^+^. Therefore, peak (1) was tentatively identified as 3-(4-hydroxy-3,5-dimethoxyphenyl)−2-propenoic acid^23^.

A peak at *m/z* 237 [M-H-C_7_H_6_O_2_]^−^ and *m/z* 193 [M-H-C_7_H_6_O_2_-COO]^−^ was observed in **Peak (2)** (Fig. 6b) [Rt 1.60 min, [M-H]^−^ at m/z 359.0726 (C_18_H_15_O_8_)^−^], which revealed the natural cleavage of the intact carboxyl residue (−44 Da). Additionally, an intense fragment at m/z 165 [M-H-C_7_H_6_O_2_-COO-CO]^−^was observed, which indicated the loss of the CO group (− 28 Da). Thus, peak (2) was hypothesized to be rosmarinic acid, a naturally occurring polyphenol molecule having anti-inflammatory and antioxidant qualities^24–26^.

**Peak (3)** (Fig. 6c) [Rt 8.44 min, [M + H]^+^ at m/z 387.1291 (C_17_H_23_O_10_)^+^] displaying distinctive fragment ions at m/z 225 and 207 corresponding to [M + H-hexoside]^+^ and [M + H-hexoside-H_2_O]^+^, respectively, revealing the aglycone parts, which are [sinapic acid + H]^+^, which are produced by the natural cleavage of intact hexoside residue (−162 Da) attached via *O*-glycosylation and water molecules (18 Da). As a result, peak (3) was hypothesized to be sinapic acid-*O*-hexoside. The cinnamate ester 1-*O*-sinapoyl-*β*-D-glucose is a glucosyl hydroxycinnamic acid that is produced via formal condensation of the anomeric hydroxy group of *β*-D-glucose with the carboxy group of trans-sinapic acid^27^.

##### Flavonoid derivatives

The primary component of flavonoids is three-ring diphenyl propane (C_6_C_3_C_6_). The glycosidic linkages are divided into *O*- and/or *C*-glycosides. It would be possible to determine the kind and location of sugar attachment in *O*- and *C*-glycosides using LC-ESI-MS/MS fragmentation patterns. Flavonoids-*O*-glycosides are sugar moiety-attached hydroxyl groups; alternatively, the anomeric carbon of the sugar part is directly connected to the aglycone component, usually at the C-6 or C-8 position (flavonoid *C*-glycosides). In *O*-glycosides, the loss of 162, 132, and 146 Da, would indicate the loss of the *O*-sugar moiety, which is represented by the *O*-hexoses, *O*-pentoses, and *O*-deoxyhexoses, respectively, due to the cleavage of the glycosidic link. *C*-glycosides, on the other hand, would exhibit inter-glycosidic cleavage of the sugar component. A characteristic fragmentation pattern of *C*-glycosides is the loss of water (−18 Da) in addition to the cross-ring cleavages of sugar units at ^0–2^X° [(O-C_1_ and C_2_-C_3_)] and ^0–3^X° [(O-C_1_ and C_3_-C_4_)] of sugar units; *C-*hexoses showed fragment [M-120/90]^+/−^, and *C-*pentoses [M-90/60]^+/−^.

##### Flavonols

The flavonol class, which comprises 15 metabolites, is primarily prevalent in the current investigation of the aerial portions of *F. arabica* L. The primary aglycones found in the identified metabolites were myricetin, kaempferol, quercetin, rhamnetin, and isorhamnetin (Table 2; Figs. 5 and 6).

Flavonol [M + H]^+^ product ions fragmentation pathway dehydrates to [M + H-H_2_O]^+^, then there are two losses of CO^−^ [M + H-H_2_O-CO]^+^ and [M + H-H_2_O-2CO]^+^. Flavonol also showed these CO losses, [M + H-CO]^+^ and [M + H-2CO]^+^. Moreover, the protonated molecules produce RDA fragments (^0,2^A^+^, ^0,2^B^+^ and ^1,3^A^+^, ^1,3^B^+^) that were created when the C-ring was broken down by the corresponding cleavage of bonds 0, 2 and 1,3.

##### Isorhamnetin derivatives

**Peak (14)** appeared at positive and negative ionization modes; Rt, 23.41 min, [M + H]^+^ at *m/z* 317.0645 (C_16_H_13_O_7_)^+^] showed a base peak fragment ion at *m/z* 302 [M + H-CH3]^+^ indicating the loss of one methyl group (−15 Da) (Fig. 6d). In addition, In negative ion mode, [M-H]^−^ at *m/z* 315.0708 (C_16_H_11_O_7_)^−^] showed ^1,3^A^−^ cleavage fragment ion at *m/z* 152 [M-H-C_9_H_7_O_3_]^−^ and ^1,3^B^−^*m/z* 163 [M-H-C_7_H_4_O_4_]^−^ followed by loss of COO group (−44 Da) to give intense fragment at *m/z* 108 [M-H-C_9_H_7_O_3_- COO]^−^ (Fig. 6e) so peak (14) was tentatively identified as 3’-methoxy-4’,5,7-trihydroxyflavonol or isorhamnetin^24,28^.

**Peak (8)** also appeared in both negative and positive ionization modes at Rt 9.54 min, [M-H]^−^ at *m/z* 623.1610 (C_28_H_31_O_16_)^−^ which exhibited the common fragmentation pattern of loss Rhamnose (146) and glucoside fragment (162) to give *m/z* 315 [M-H-rutinoside]^−^ (Fig. 6f), and [M + H]^+^ at *m/z* 625.1696 (C_28_H_33_O_16_)^+^ displayed MS/MS spectrum showing significant peaks at *m/z* 479 [M + H-146]^+^ and *m/z* 317 [M + H-146–162]^+^ (Fig. 6g). Therefore, peak (8) was tentatively identified as isorhamnetin-3-*O*-rutinoside^24,29^.

##### Kaempferol derivatives

**Peak (16)** appeared in both negative and positive ionization modes at Rt 23.60 min, [M-H]^−^ at *m/z* 299.0516 (C_16_H_11_O_6_)^−^ which exhibited the common fragmentation pattern of the aglycone to give *m/z* 284 [M-2 H-CH_3_]^−^ (Fig. 6n), and [M + H]^+^ at *m/z* 301.0665 (C_16_H_13_O_6_)^+^ displayed MS/MS spectrum showing significant peaks at *m/z* 286 [M + H-15]^+^ (Fig. 6o).Therefore, peak (16) was tentatively identified as 4`-methoxyKaempferol^24,28,30^.

**Peak (13)** appeared in both negative and positive ionization modes at Rt 10.10 min, [M-H]^−^ at *m/z* 447.0926 (C_21_H_19_O_11_)^−^ which exhibited the common fragmentation pattern of the aglycone to give *m/z* 284 [M-2 H-Glc]^−^ (Fig. 6m), and [M + H]^+^ at *m/z* 499.1800 (C_21_H_21_O_11_)^+^ displayed MS/MS spectrum showing significant peaks at *m/z* 287 [M + H-162]^+^ (Fig. 6l).Thus, peak (10) was tentatively identified as kaempferol-3-*O*-glucoside^31^.

**Peak (12)** appeared in both negative and positive ionization modes at Rt 10.05 min, [M-H]^−^ at *m/z* 593.1484 (C_30_H_25_O_13_)^−^ which exhibited the common fragmentation pattern of loss coumaroyl moiety (146) and glucoside fragment (162) to give *m/z* 285 [M-H-coumaroylglucoside]^−^ (Fig. 6i), besides, [M + H]^+^ at *m/z* 595.1382 (C_30_H_27_O_13_)^+^ displayed MS/MS spectrum showing significant peaks at *m/z* 449 [M + H-146]^+^ and *m/z* 287 [M + H-146–162]^+^ (Fig. 6h).Therefore, peak (12) was tentatively identified as Kaempferol-3-*O*-(6-*p*-coumaroyl)-glucoside^28^.

**Peak (7)** appeared in negative ionization mode at Rt 9.42 min, [M-H]^−^ at *m/z* 417.0822 (C_20_H_17_O_10_)^−^ which exhibited the common fragmentation pattern of loss arabinose (−132) *m/z* 285 [M-H-Ara]^−^. Therefore, peak (7) was tentatively identified as kaempferol-3-*O-α*-L-arabinoside^24,32^.

**Peak (6)** appeared in both negative and positive ionization modes at Rt 9.18 min, [M-H]^−^ at *m/z* 739.2067 (C_33_H_39_O_19_)^−^ which exhibited the common fragmentation pattern of the aglycone to give *m/z* 285 [M-H-robinoside-Rha]^−^ (Fig. 6k), and [M + H]^+^ at *m/z* 741.2571 (C_33_H_41_O_19_)^+^ displayed MS/MS spectrum showing significant peaks at *m/z* 595 [M + H-146]^+^ and *m/z* 449 [M + H-146-146]^+^ and *m/z* 287 [M + H-146-146-162]^+^ (Fig. 6j).Therefore, peak (9) was tentatively identified as kaempferol-3-*O*-robinoside-7-*O*-rhamnoside^24,33^.

##### Quercetin derivatives

**Peak (18)** appeared in positive ionization mode at Rt 24.03 min, [M + H]^+^ at *m/z* 303.0809 (C_15_H_11_O_7_)^+^ which exhibited the common fragmentation pattern of loss H_2_O (−18) *m/z* 285 [M + H-H_2_O]^+^, *m/z* 257 [M + H-H_2_O-CO]^+^ and *m/z* 229 [M + H-H_2_O-2CO]^+^. In addition, fragments ^**0,2**^A^**+**^, ^**0,2**^B^**+**^*m/z* 165, 138, respectively (Fig. 6p). Therefore, peak (18) was tentatively identified as quercetin^24,34^.

**Peak (10)** appeared in both negative and positive ionization modes at Rt 9.68 min, [M-H]^−^ at *m/z* 625.1367 (C_27_H_29_O_17_)^−^ which exhibited the common fragmentation pattern of loss glucoside (− 162) *m/z* 463 [M-H-Glc]^−^ and another glucoside fragment (− 162) to give *m/z* 300 [M-2 H-2Glc]^−^ (Fig. 6s), and [M + H]^+^ at *m/z* 627.1559 (C_27_H_31_O_17_)^+^ displayed MS/MS spectrum showing significant peaks at *m/z* 465 [M + H-162]^+^ and *m/z* 303 [M + H-162-162]^+^ (Fig. 6t).Therefore, peak (10) was tentatively identified as quercetin-3,4’-*O*-di-*β*-glucopyranoside^24,29^.

**Peak (9)** (Fig. 6q) appeared in negative ionization mode at Rt 9.55 min, [M-H]^−^ at *m/z* 595.1295 (C_26_H_27_O_16_)^−^ which exhibited the common fragmentation pattern of loss arabinose (− 132) *m/z* 463 [M-H-Ara]^−^, and glucoside fragment (− 162) to give *m/z* 300 [M-2 H-Ara-Glc]^−^ and 271 [M-2 H-Ara-Glc-CO]^−^. Therefore, peak (9) was tentatively identified as quercetin-3-*O*-arabinoglucoside^31^.

**Peak (5)** (Fig. 6r) appeared in positive ionization mode at Rt 9.11 min, [M + H]^+^ at *m/z* 597.1415 (C_26_H_29_O_16_)^+^ which exhibited the common fragmentation pattern of loss glucoside (− 162) *m/z* 435 [M + H-Glc]^+^, and arabinoside fragment (− 132) to give *m/z* 303 [M + H-Glc-Ara]^+^. Therefore, peak (5) was tentatively identified as quercetin-*O*-pentoside-*O*-hexoside^31^.

##### Myricetin derivatives

**Peak (17)** (Fig. 6u) appeared in negative ionization mode at Rt 23.62 min, [M-H]^−^ at *m/z* 317.0536 (C_15_H_9_O_7_)^−^ which exhibited the common fragmentation pattern of 225 [M-H-2H_2_O-2CO]^−^ and 164 [^0,2^A^−^]^**−**^. Therefore, peak (17) was tentatively identified as myricetin^24,28^.

**Peak (11)** (Fig. 6v) appeared in positive ionization mode at Rt 9.87 min, [M + H]^+^ at *m/z* 509.1251 (C_23_H_25_O_13_)^+^ which exhibited a fragmentation pattern of 347 [M + H-Glc]^+^ and 332 [M + H-Glc-CH_3_]^+^. Therefore, peak (11) was tentatively identified as syringetin-3-*O*-glucoside^31^.

##### Gossypetin derivative

**Peak (4)** (Fig. 6w) appeared in negative ionization mode at Rt 8.99 min, [M-H]^−^ at *m/z* 479.1201 (C_21_H_19_O_13_)^−^ which exhibited fragmentation patterns of 461 [M-H-H_2_O]^−^, 433 [M-H-H_2_O-CO]^−^, 317 [M-H-Glc]^−^. Therefore, peak (4) was tentatively identified as gossypin^28^.

##### Flavanol

**Peak (19)** (Fig. 6x) appeared in positive ionization mode at Rt 21.44 min, [M + H]^+^ at *m/z* 305.0661 (C_15_H_13_O_7_)^+^ which exhibited a fragmentation pattern of 287 [M + H-H_2_O]^+^, 212 [M-2H_2_O-2CO]^+^, 185 [M + H-2H_2_O-3CO]^+^ and 139 [M + H-3H_2_O-4CO]^+^. Therefore, peak (19) was tentatively identified as (±)taxifolin^28^ (Table 2; Figs. 5 and 6).

##### Flavanone

**Fig. 7:**
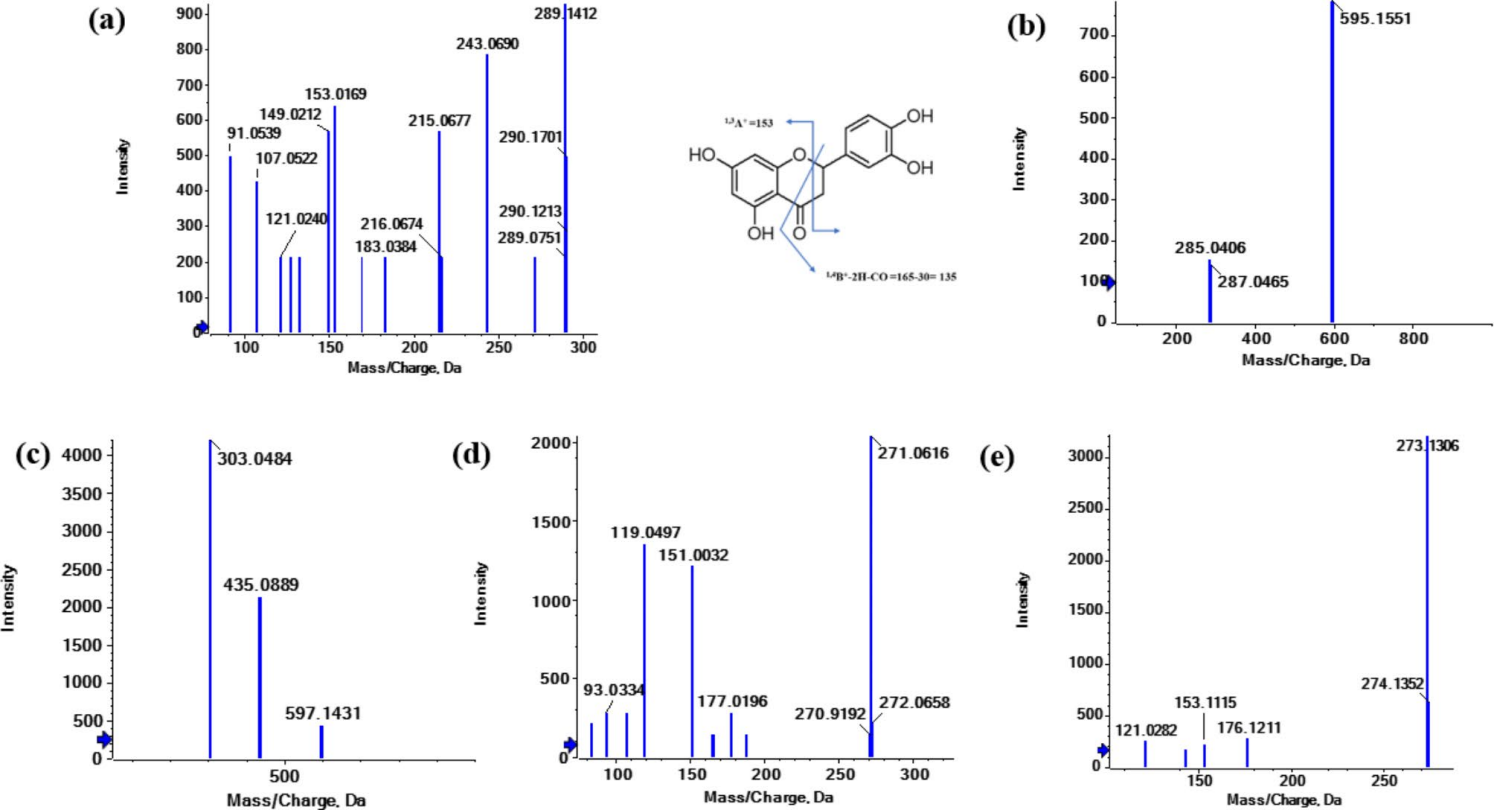
Fragmentation figures of the main identified flavanone compounds in *Fagonia arabica* L. hydro-methanolic extract (**a**) MS/MS spectrum of eriodictyol aglycone in positive ion mode, (**b**) MS/MS spectrum of eriodictyol-7-*O*-neohesperidoside in negative mode, (**c**) MS/MS spectrum of eriodictyol-*O*-hexoside-*O-*pentaside in positive ion mode, (**d**), (**e**) MS/MS spectrum of naringenin aglycone at both negative and positive mode, respectively.

Flavanone [M + H]^+^ product ions dehydrated to [M + H-H_2_O]^+^. flavanone itself undergoes cleavages to yield the ^1,3^A^+^ and ^1,4^B^+^ ions. ^1,4^B^+^ fragment ion mainly appears as [^1,4^B^+^−2 H-CO]^+^ (Table 2; Figs. 5 and 7).

##### Eriodictyol aglycone

**Peak (22)** (Fig. 7a) appeared in positive ion mode at Rt 23.91 min, [M + H]^+^ at m/z 289.0751 (C_15_H_13_O_6_)^+^, which exhibited the fragmentation patterns of m/z 271 [M + H-H_2_O]^+^, m/z 243 [M + H-H_2_O-CO]^+^, m/z 215 [M + H-H_2_O-2CO]^+^, m/z 153 [^1,3^A]^+^ and m/z 135 [^1,4^B^+^−2 H-CO]^+^. Therefore, peak (22) was tentatively identified as eriodictyol^28^.

**Peak (21)** (Fig. 7c) appeared in positive ionization mode at Rt 9.10 min as [M + H]^+^ at *m/z* 597.1431 (C_27_H_33_O_15_)^+^ which exhibited the common fragmentation pattern of loss hexoside moeity 435 [M + H- 162]^+^ followed by loss of pentaside part at 303 [M + H-162-132]^+^. Therefore, peak (21) was tentatively identified as eriodictyol-*O*-hexoside-*O-*pentaside^30^.

**Peak (20)** (Fig. 7b) appeared in negative ion mode at Rt 7.02 min, [M-H]^−^ at m/z 595.1551 (C_27_H_31_O_15_)^−^, which exhibited the fragmentation patterns of m/z 287 [M-H-146–162]^−^ = [M-H-Rha-Glc]^−^. Therefore, peak (20) was tentatively identified as eriodictyol-7-*O*-neohesperidoside^30^.

##### Naringenin aglycone

**Peak (23)**, at Rt 24.97, [M + H]^+^ for (C_15_H_13_O_5_)^+^ at m/z 273.1306 showed the main fragmentation pattern of m/z 153 [^1,3^A]^+^ and m/z 121 [^1,4^B^+^−2 H-CO]^+^ (Fig. 7e**).** While, [M-H]^−^ at m/z 271.0616 showed m/z 151 [^1,3^A]^−^ and m/z 119 [^1,4^B^−^−2 H-CO]^−^ (Fig. 7d). Therefore, peak (23) was tentatively identified as naringenin aglycone^24,30^.

##### Flavones

**Fig. 8:**
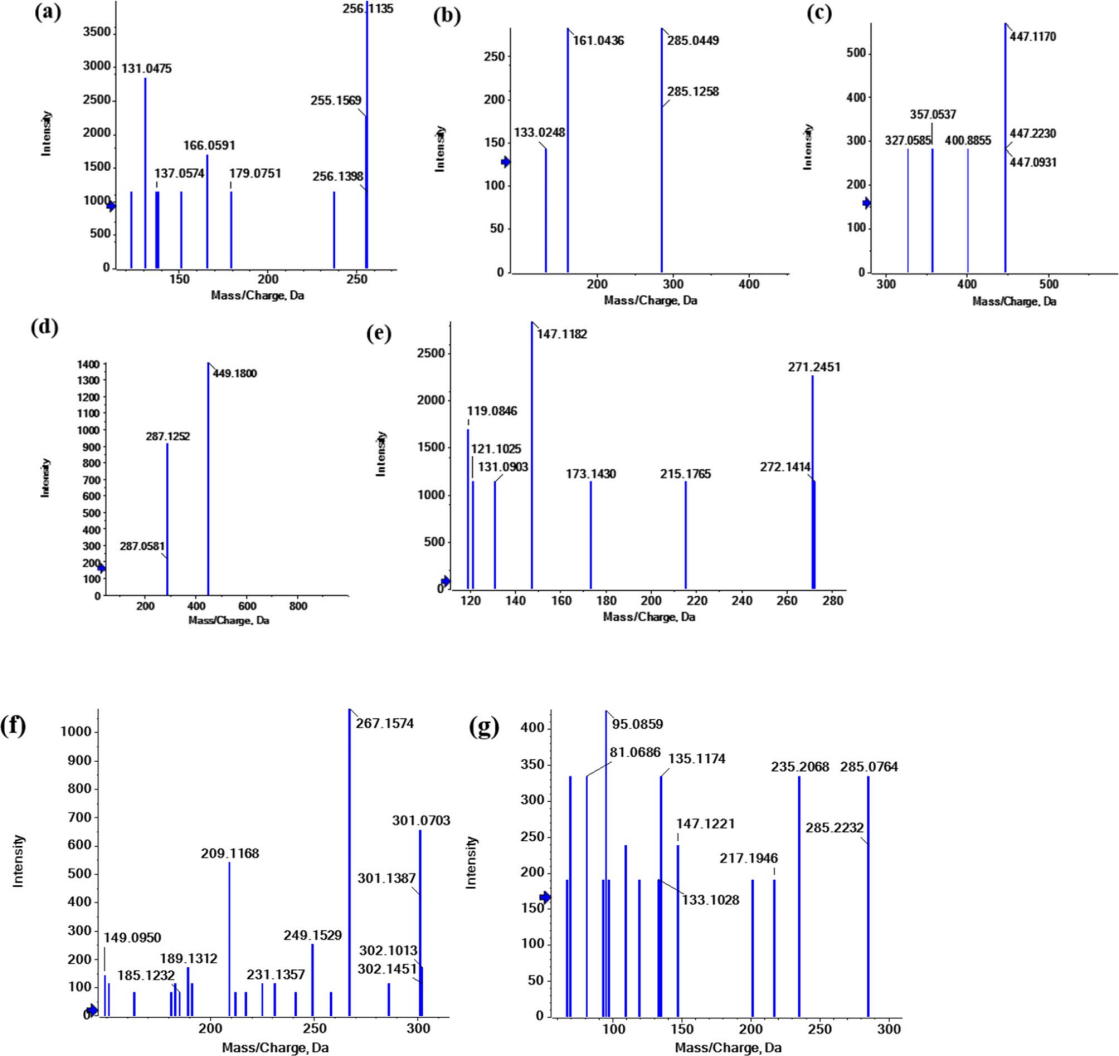
Fragmentation figures of the identified flavone and isoflavone compounds in *Fagonia arabica* L. hydro-methanolic extract (**a**) MS/MS spectrum of daidzein aglycone in positive ion mode, (**b**) MS/MS spectrum of luteolin aglycone in negative ion mode, (**c**), (**d**) MS/MS spectrum of luteolin-8-*C*-glucoside at both negative and positive mode, respectively, (**e**) MS/MS spectrum of apigenin aglycone in positive ion mode, (**f**) MS/MS spectrum of diosmetin aglycone in positive ion mode, (**g**) MS/MS spectrum of acacetin aglycone in positive ion mode.

The flavone class comprises 8 metabolites in the current investigation of the aerial portions of *F. arabica* L. The primary aglycones found in the identified metabolites were luteolin, apigenin, diosmetin and acacetin (Table 2; Figs. 5 and 8).

##### Luteolin derivatives

**Peak (28)** (Fig. 8b) appeared in negative ion mode at Rt 21.56 min, 285.0449 [M-H]^−^, (C_15_H_9_O_6_)^−^, which exhibited the fragmentation patterns of m/z 161 [^0,4^B-H_2_O]^−^ and 133 [^1,3^B]^−^. Therefore, peak (28) was tentatively identified as luteolin aglycone^24,30,35^.

**Peak (27)** appeared in positive and negative ion modes at Rt 9.93 min, [M + H]^+^ at *m/z* 449.1800 (C_21_H_21_O_11_)^+^] displayed the common fragmentation pattern of luteolin-mono-*O*-glycoside exhibiting indicative fragments at *m/z* 287 [M + H-Glc]^+^ (Fig. 8d) and [M-H]^−^ at *m/z* 447.0931 (C_21_H_19_O_11_)^−^ and displayed the common fragmentation pattern of luteolin-mono-*C*-glycoside exhibiting indicative fragments at *m/z* 357 [M-H-90]^−^[Ag + 71]^−^ and *m/z* 327 [M-H-120]^−^[Ag + 41]^−^ (Fig. 8c). Therefore, peak (27) was tentatively identified as luteolin-8-*C*-glucoside^24,30,35^.

##### Apigenin derivatives

**Peak (29)** (Fig. 8e) appeared at Rt 22.94 in positive ionization mode at 271.2451 [M + H]^+^ corresponding to (C_15_H_11_O_5_)^+^ and showed a fragmentation pattern of 215 [M + H-2CO]^+^, 173 [M + H-2CO-CH_2_CO]^+^, 121 [^0,2^B^+^] and 119 [^1,3^B^+^]. Therefore, peak (29) was tentatively identified as apigenin aglycone^24,36^.

**Peak (25)** appeared at Rt 8.70 in positive ionization mode at 433.11292 [M + H]^+^ corresponding to (C_21_H_21_O_10_)^+^ and showed a fragmentation pattern of 343.1017 [M + H-90]^+^ and 313.0681 [M + H-120]^+^. Therefore, peak (29) was tentatively identified as apigenin 8-*C*-glucoside^24,37^.

**Peak (24)** appeared at Rt 8.44 in positive ionization mode at 595.16577 [M + H]^+^ corresponding to (C_27_H_31_O_15_)^+^ and showed a fragmentation pattern of 433.1402 [M + H-Glc]^+^ and 313.0711 [M + H-Glc-120]^+^. Therefore, peak (24) was tentatively identified as apigenin-6-*C*-glucoside − 7-*O*-glucoside^[24,36^..

##### Diosmetin derivatives

**Peak (30)** (Fig. 8f) appeared at Rt 23.47 in positive ionization mode at 301.0703 [M + H]^+^ corresponding to (C_16_H_13_O_6_)^+^ and showed a fragmentation pattern of 286 [M + H-15]^+^, 267 [M-CH_3_-H_2_O]^+^, 249 [M-CH_3_−2H_2_O]^+^, 191[^0,4^B^+^−2 H] and 149 [^1,3^B^+^]. Therefore, peak (30) was tentatively identified as diosmetin^38^.

**Peak (26)** appeared at Rt 9.60 in positive ionization mode at 609.1796 [M + H]^+^ corresponding to (C_28_H_33_O_15_)^+^ and showed a fragmentation pattern of 463.1213 [M + H-Rha]^+^ and 301.0701 [M + H-Rha-Glc]^+^. Therefore, peak (26) was tentatively identified as diosmin^38^.

##### Acacetin aglycone

**Peak (31)** (Fig. 8g) appeared at Rt 23.74 in positive ionization mode at 285.0764 [M + H]^+^ corresponding to (C_16_H_13_O_5_)^+^ and showed a fragmentation pattern of 235.2064 [M + H-CH_3_-H_2_O-OH]^+^ and 217.1946 [M + H-CH_3_−2H_2_O-OH]^+^. Therefore, peak (31) was tentatively identified as acacetin^24,30,39^.

##### Isoflavones

Isoflavones exhibited a series of consistent neutral losses in the MS spectra, including 28 Da, 44 Da, 56 Da, 72 Da, and 84 Da, which correspond to the loss of CO, CO_2_, 2CO, CO + CO_2_, and 3CO, respectively. In addition to these common neutral losses, isoflavones undergo Retro-Diels–Alder (RDA) fragmentation, producing the ^0,4^B^+^-H_2_O and ^1,3^A^+^ ions. A complete loss of the B ring, resulting in the [M-B ring-CO]^+^ ion, is also frequently observed (Table 2; Figs. 5 and 8).

##### Daidzein aglycone

**Peak (33)** (Fig. 8a) eluted at Rt 22.89 and appeared at [M + H]^+^ = 255.1569 corresponding to (C_15_H_11_O_4_)^+^ and showed a fragmentation pattern of 237 [M + H-H_2_O]^+^, 179 [M + H-H_2_O-2 H-2CO]^+^ and 137 [^1,3^A^+^]. Therefore, peak (33) was tentatively identified as daidzein aglycone^28^.

**Peak (32)** eluted at Rt 9.38 and appeared at [M + H]^+^ = 417.2717 corresponding to (C_21_H_21_O_9_)^+^ and showed a fragmentation pattern of 399 [M + H-H_2_O]^+^, 343 [M + H-H_2_O-2CO]^+^, 325 [M + H-2H_2_O-2CO]^+^, 327 [M + H-90]^+^, 297 [M + H-120]^+^, 119 [^1,3^B^+^]. Therefore, peak (33) was tentatively identified as daidzein-8-*C*-glucoside^28^.

##### Chalcones

**Fig. 9:**
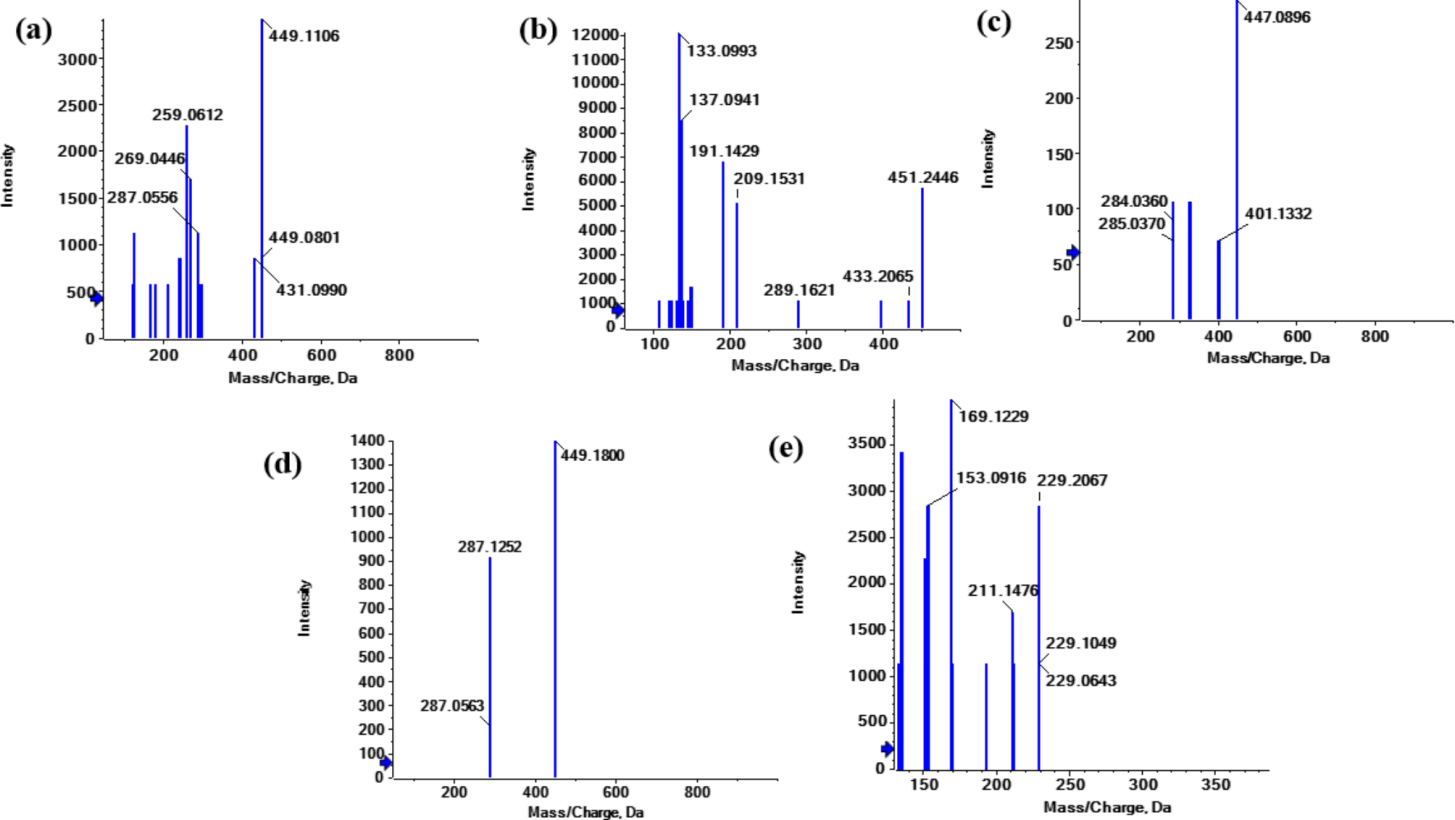
Fragmentation figures of the identified chalcones, aurone *O*-glycosides and stilbenes in *Fagonia arabica* L. hydro-methanolic extract, (**a**,**b**) MS/MS spectrum of okanin-4’-*O*-glucoside at both negative and positive mode, respectively, (**c**,**d**) MS/MS spectrum of maritimetin-6-*O*-glucoside at both negative and positive mode, respectively, (**e**) MS/MS spectrum of resveratrol in positive ion mode.

**Peak (34)** (Table 2; Figs. 5 and 9) appeared in both negative and positive ionization modes at Rt 1.22 min, (a) [M-H]^−^ at *m/z* 449.1106 (C_21_H_21_O_11_)^−^ which exhibited the common fragmentation pattern of loss glucoside (−162) *m/z* 287 [M-H-Glc]^−^ and another loss of water fragment (−18) to give *m/z* 431 [M-H-H_2_O]^−^ and *m/z* 269 [M-H-Glc-H_2_O]^−^ (Fig. 9a) (b) [M + H]^+^ at *m/z* 451.2446 (C_27_H_31_O_17_)^+^ displayed MS/MS spectrum showing significant peaks at *m/z* 289 [M + H-162]^+^ and *m/z* 433 [M + H-H_2_O]^+^ also showed [A + H]^+^ =[M + H-Glc-C_7_H_5_O_4_] at *m/z* 137 (Fig. 9b). Therefore, peak (34) was tentatively identified as okanin-4’-*O*-glucoside^35^.

##### Aurone *O*-glycosides

**Peak (35)** (Table 2; Figs. 5 and 9) appeared in both negative and positive ionization modes at Rt 7.11 min, (a) [M-H]^−^ at *m/z* 447.0896 (C_21_H_19_O_11_)^−^ which exhibited the common fragmentation pattern of loss 2 molecules of water at *m/z* 401 [M-H-2H_2_O]^−^ and loss of glucoside moiety (−162) *m/z* 285 [M-H-Glc]^−^ (Fig. 9c), (b) [M + H]^+^ at *m/z* 449.1800 (C_21_H_21_O_11_)^+^ displayed MS/MS spectrum showing significant peak at *m/z* 287 [M + H-162]^+^ (Fig. 9d). Therefore, peak (35) was tentatively identified as maritimetin-6-*O*-glucoside^39^.

##### Stilbenes

**Peak (36)** (Table 2; Figs. 5 and 9e) eluted at Rt 9.36 and appeared at [M + H]^+^=229.0643 corresponding to (C_14_H_13_O_3_)^+^ and showed a fragmentation pattern of 211 [M + H-H_2_O]^+^, 193 [M + H-2H_2_O]^+^. Therefore, peak (36) was tentatively identified as resveratrol^40^.

##### Anthocyanins

**Fig. 10:**
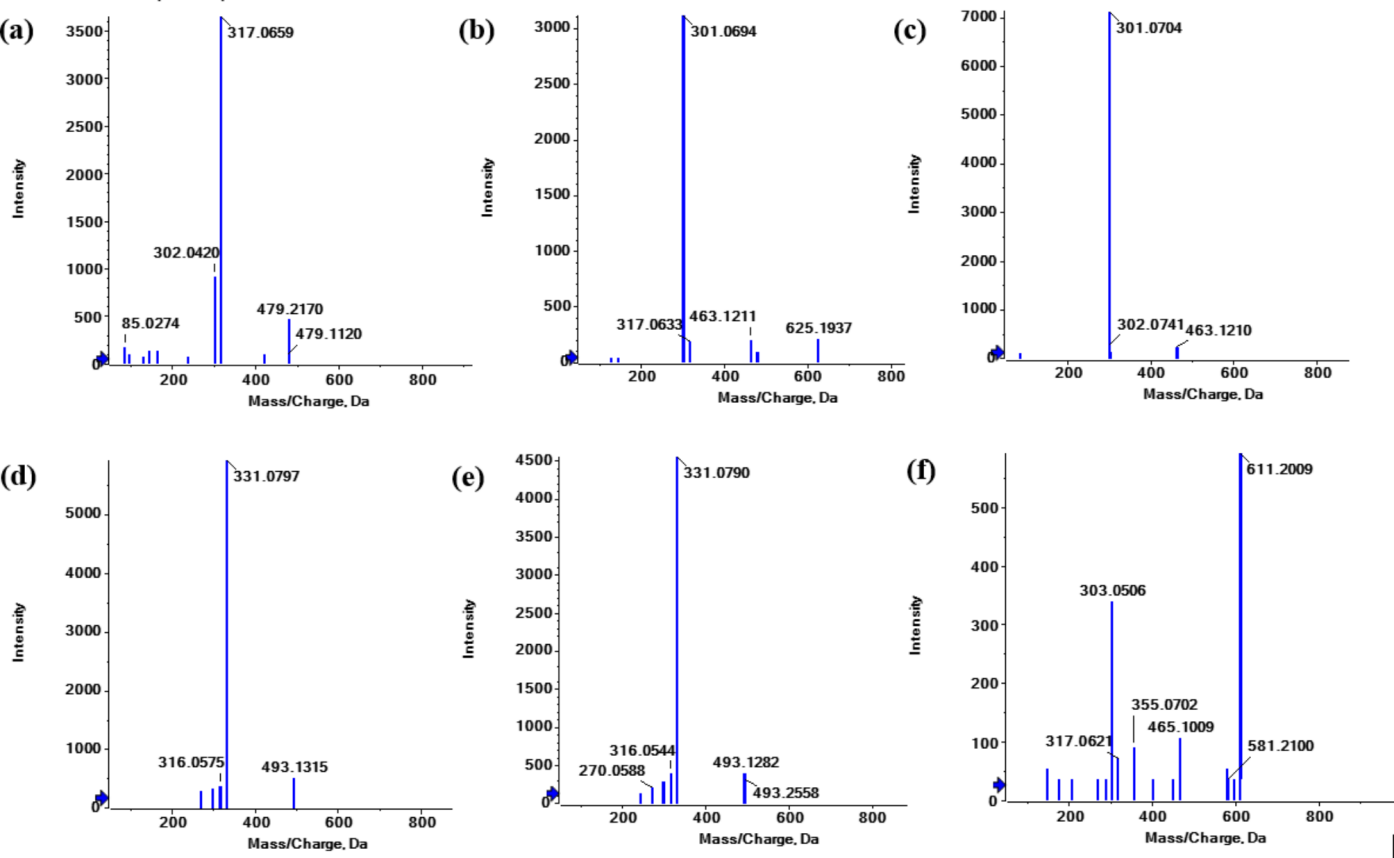
Fragmentation figures of the identified anthocyanins in *Fagonia arabica* L. hydro-methanolic extract in positive ionization mode (**a**) MS/MS spectrum of petunidin-3-*O*-*β*-glucopyranoside (**b**) MS/MS spectrum of peonidin-3,5-*O*-di-*β*-glucopyranoside, (**c**) MS/MS spectrum of peonidine-3-*O*-glucoside, (**d**) MS/MS spectrum of malvidin-3-*O*-glucoside, (**e**) MS/MS spectrum of malvidin-3-*O*-galactoside, (**f**) MS/MS spectrum of delphinidin-3-*O*-(6’’-*O*-*α*-rhamnopyranosyl-*β*-glucopyranoside).

The anthocyanin class comprises 6 metabolites in the current investigation of the aerial portions of *F. arabica* L (Table 2; Figs. 5 and 10). The reported fragments are [M-H_2_O]^+^, [M-CO]^+^, [M-CO-H_2_O]^+^, [M-2CO]^+^ and [M-CH_3_]^+^.

**Peak (37)** (Fig. 10f) eluted at Rt 9.48 and appeared at 611.1590 [M]^+^, corresponding to (C_27_H_31_O_16_)^+^ and showed a fragmentation pattern of 465 [M-146]^+^ and 303 [M-146–162]^+^. Therefore, peak (37) was tentatively identified as delphinidin-3-*O*-(6’’-*O*-*α*-rhamnopyranosyl-*β*-glucopyranoside)^24,41^.

**Peak (38)** (Fig. 10b) eluted at Rt 9.54 and appeared at 625.193 [M]^+^, corresponding to (C_28_H_33_O_16_)^+^ and showed a fragmentation pattern of 463 [M-162]^+^ and 301 [M-2Glc]^+^. Therefore, peak (38) was tentatively identified as peonidin-3,5-*O*-di-*β*-glucopyranoside^30,42^.

**Peak (39)** (Fig. 10c) eluted at Rt 10.13 and appeared at 463.1210 [M]^+^, corresponding to (C_22_H_23_O_11_)^+^ and showed a fragmentation pattern of 301 [M-162]^+^. Therefore, peak (39) was tentatively identified as peonidine-3-*O*-glucoside^30,42^.

**Peak (40)** (Fig. 10a) eluted at Rt 10.21 and appeared at 479.1120 [M]^+^, corresponding to (C_22_H_23_O_12_)^+^ and showed a fragmentation pattern of 317 [M-162]^+^ and 302 [M-162-CH_3_]^+^. Therefore, peak (40) was tentatively identified as petunidin-3-*O*-*β*-glucopyranoside^30,42^.

**Peak (41)** (Fig. 10e) eluted at Rt 10.47 and appeared at 493.1282 [M]^+^, corresponding to (C_23_H_25_O_12_)^+^ and showed a fragmentation pattern of 331 [M-162]^+^, 316 [M-162-CH_3_]^+^ and 270 [M-162-CH_3_-H_2_O-CO]^+^. Therefore, peak (41) was tentatively identified as malvidin-3-*O*-galactoside^24,43^.

**Peak (42)** (Fig. 10d) eluted at Rt 10.54 and appeared at 493.1315 [M]^+^, corresponding to (C_23_H_25_O_12_)^+^ and showed a fragmentation pattern of 331 [M-162]^+^ and 316 [M-162-CH_3_]^+^. Therefore, peak (42) was tentatively identified as malvidin-3-*O*-glucoside^30,42,43^.

**Table 2:** Metabolites identified by UPLC-qTOF-MS/MS analysis of total hydro-methanolic extract of *F. arabica* L. aerial parts.

Peak no.	Rt (min)	[M-H]^−^	[M + H]^+^	Formula	MS^*n*^ (m/z) negative mode	MS^*n*^ (m/z) positive mode	Metabolite	References
Phenolic acids (cinnamic acid derivatives)								
1	1.05	–	225.1833	C_11_H_12_O_5_	–	225, 210, 195, 177, 149, 133	3-(4-hydroxy-3,5-dimethoxyphenyl)−2-propenoic acid	^23^
2	1.60	359.0726	–	C_18_H_16_O_8_	237, 193, 165		Rosmarinic acid	^24–26^
3	8.44	–	387.1291	C_17_H_22_O_10_	–	225, 207	1-*O*-sinapoyl-*β*-D-glucose	^27^
Flavonols								
4	8.99	479.1201	–	C_21_H_20_O_13_	461, 433, 317	–	Gossypin	^28^
5	9.11	–	597.1415	C_26_H_28_O_16_	–	435, 303	Quercetin-*O*-pentoside-*O*-hexoside	^31^
6	9.18	739.2067	741.2571	C_33_H_40_O_19_	285	595, 449, 287	Kaempferol-3-*O*-robinoside-7-*O*-rhamnoside	^24,33^
7	9.42	417.0822	–	C_20_H_18_O_10_	285	–	Kaempferol-3-*O-α*-L-arabinoside	^24,32^
8	9.54	623.1610	625.1696	C_28_H_32_O_16_	315	479, 317	Isorhamnetin-3-*O*-rutinoside	^24,29^
9	9.55	595.1295	–	C_26_H_28_O_16_	463, 300, 271	–	Quercetin-3-*O*-arabinoglucoside	^31^
10	9.68	625.1367	627.1559	C_27_H_30_O_17_	463,300	465, 303	Quercetin-3,4’-*O*-di-*β*-glucopyranoside	^24,29^
11	9.87	–	509.1251	C_23_H_24_O_13_	–	347, 332	Syringetin-3-*O*-glucoside	^31^
12	10.05	593.1484	595.1382	C_30_H_26_O_13_	285	449, 287	Kaempferol-3-*O*-(6-*p*-coumaroyl)-glucoside	^28^
13	10.10	447.0926	449.1800	C_21_H_20_O_11_	284	287	Kaempferol-3-*O*-glucoside	^31^
14	23.41	315.0708	317.0645	C_16_H_12_O_7_	163, 152, 108	302	3’-methoxy-4’,5,7-trihydroxyflavonol	^24,28^
15	23.50	315.0708	317.0645	C_16_H_12_O_7_	152	302	3, 3’, 4’, 5-tetrahydroxy-7-methoxyflavone [Rhamnetin]	^24^
16	23.60	299.0516	301.0665	C_16_H_12_O_6_	284, 255, 227	286, 258, 167	3, 5, 7-trihydroxy-4’-methoxyflavone [4`-methoxyKaempferol]	^24,28,30^
17	23.62	317.0536	–	C_15_H_10_O_8_	225, 164	–	Myricetin	^24,28^
18	24.03	–	303.0809	C_15_H_10_O_7_	–	285, 257, 229, 165, 138	Quercetin	^24,34^
Flavanol								
19	21.44	–	305.0661	C_15_H_12_O_7_	–	287, 212, 185, 139	(±)-Taxifolin	^28^
Flavanones								
20	7.02	595.1551	–	C_27_H_32_O_15_	287	–	Eriodictyol-7-*O*-neohesperidoside	^30^
21	9.10	–	597.1431	C_27_H_32_O_15_	–	435, 303	Eriodictyol-*O*-hexoside-*O-*pentaside	^30^
22	23.91	287.0635	289.0751	C_15_H_12_O_6_	154, 136	271, 243, 215, 135	3’, 4’, 5, 7-tetrahydroxyflavanone	^28^
23	24.97	271.0616	273.1306	C_15_H_12_O_5_	151, 119	176, 153, 121	Naringenin	^24,30^
Flavones								
24	8.44	–	595.16577	C_27_H_30_O_15_	–	433, 313	Apigenin-6-*C*-glucoside − 7-*O*-glucoside	^24,38^
25	8.70	–	433.11292	C_21_H_20_O_10_	–	343, 313	Apigenin 8-*C*-glucoside	^17,36^
26	9.60	–	609.1796	C_28_H_32_O_15_	–	463, 301	Diosmin	^38^
27	9.93	447.0931	449.1800	C_21_H_20_O_11_	357, 327	287	Luteolin-8-*C*-glucoside	^24,30,35^
28	21.56	285.0449	–	C_15_H_10_O_6_	161, 133	–	Luteolin	^24,30,35^
29	22.94	–	271.2451	C_15_H_10_O_5_	–	215, 173, 121, 119	Apigenin	^24,36^
30	23.47	–	301.0703	C_16_H_12_O_6_	–	286, 267, 249, 191, 149	Diosmetin	^38^
31	23.74	–	285.0764	C_16_H_12_O_5_	–	235, 217, 147, 135,133	Acacetin	^24,30,39^
Isoflavones								
32	9.38	–	417.2717	C_21_H_20_O_9_	–	399, 343, 325, 327, 297, 119	Daidzein-8-*C*-glucoside	^28^
33	22.89	–	255.1569	C_15_H_10_O_4_	–	237, 179, 137	Daidzein	^28^
Chalcones								
34	1.22	449.1106	451.2446	C_21_H_22_O_11_	431, 287, 269	433, 289, 137	Okanin-4’-*O*-glucoside	^35^
Aurone O-glycosides								
35	7.11	447.0896	449.1800	C_21_H_20_O_11_	401, 285	287	Maritimetin-6-*O*-glucoside	^39^
Stilbene								
36	9.36	–	229.0643	C_14_H_12_O_3_	–	211, 193, 169, 153	Resveratrol	^40^

Peak no.	Rt (min)	[M]^+^	Formula	MS^*n*^ (m/z) positive mode	Metabolite	References		
Anthocyanins								
37	9.48	611.1590	C_27_H_31_O_16_^+^	465, 303	Delphinidin-3-*O*-(6’’-*O*-*α*-rhamnopyranosyl-*β*-glucopyranoside)	^24,41^		
38	9.54	625.193	C_28_H_33_O_16_^+^	463, 301	Peonidin-3,5-*O*-di-*β*-glucopyranoside	^30,42^		
39	10.13	463.1210	C_22_H_23_O_11_^+^	301	Peonidine-3-*O*-glucoside	^30,42^		
40	10.21	479.1120	C_22_H_23_O_12_^+^	317, 302	Petunidin-3-*O*-*β*-glucopyranoside	^30,42^		
41	10.47	493.1282	C_23_H_25_O_12_^+^	331, 316, 270	Malvidin-3-*O*-galactoside	^24,43^		
42	10.54	493.1315	C_23_H_25_O_12_^+^	331, 316	Malvidin-3-*O*-glucoside	^30,42,43^		

### Biological evaluation

#### Anti-butyrylcholinesterase activity of *F. Arabica* total hydro-methanolic extract and its successive fractions

**Fig. 11:**
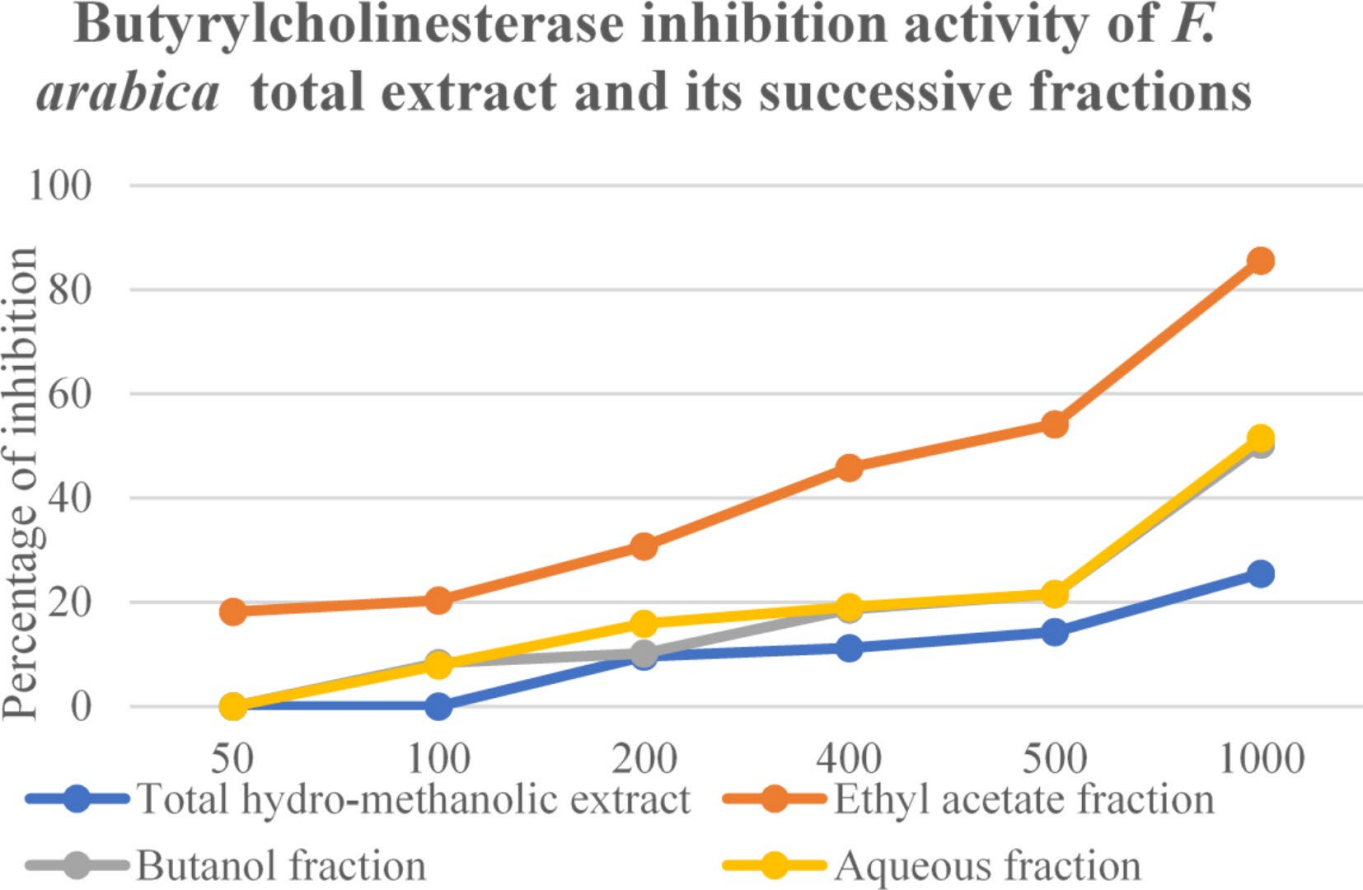
Anti-butyrylcholinesterase activity of *Fagonia arabica* L. total hydro-methanolic extract and its successive fractions.

The butyrylcholinesterase enzyme was tested in vitro to see whether the whole hydro-methanolic extract and its fractions [ethyl acetate (EtOAc), *n*-butanol (*n*-BuOH), and residual aqueous fraction] had any inhibitory effects (Fig. 11). The positive control in this case was the reference medication donepezil. According to Table 3 data, the EtOAc fraction demonstrated potential inhibitory activity against the enzyme at 0.45 ± 1.3 mg/mL, with a percentage of inhibition of 50% (IC_50_). Its efficiency was partially explained by the fact that it contains a large number of phenolic chemicals, all of which have been demonstrated to have an impact on this enzyme^7,8^. Quercetin, was shown to exhibit a notable inhibitory effect against acetylcholinesterase (AChE) with a 76.2% inhibition^2^ or IC_50_of 19.8^9^. In a different study, a wide range of phenolic acids and flavonoid derivatives, including naringin and diosmin, showed an ability to inhibit AChE and butyrylcholinesterase (BChE)^44^. Quercetin, myricetin, and luteolin reversibly inhibit human BChE^8^.

**Table 3:** IC_50_ of *Fagonia Arabica* ethyl acetate, butanol and aqueous fractions against butyrylcholinesterase.

Sample	IC_50_ (mg/mL)
*F. arabica*ethyl acetate fraction	0.45 ± 1.3
*F. arabica*butanol fraction	1.01 ± 0.88
*F. arabica*remaining aqueous fraction	0.99 ± 1.77

### Molecular docking study

A molecular modelling analysis was carried out using AutoDock Vina to clarify interactions, binding affinities, and selectivity towards butyrylcholinesterase of tentatively identified phenolic compounds inside the Acetylcholinesterase and butyrylcholinesterase enzymes (Table S1).

**Fig. 12:**
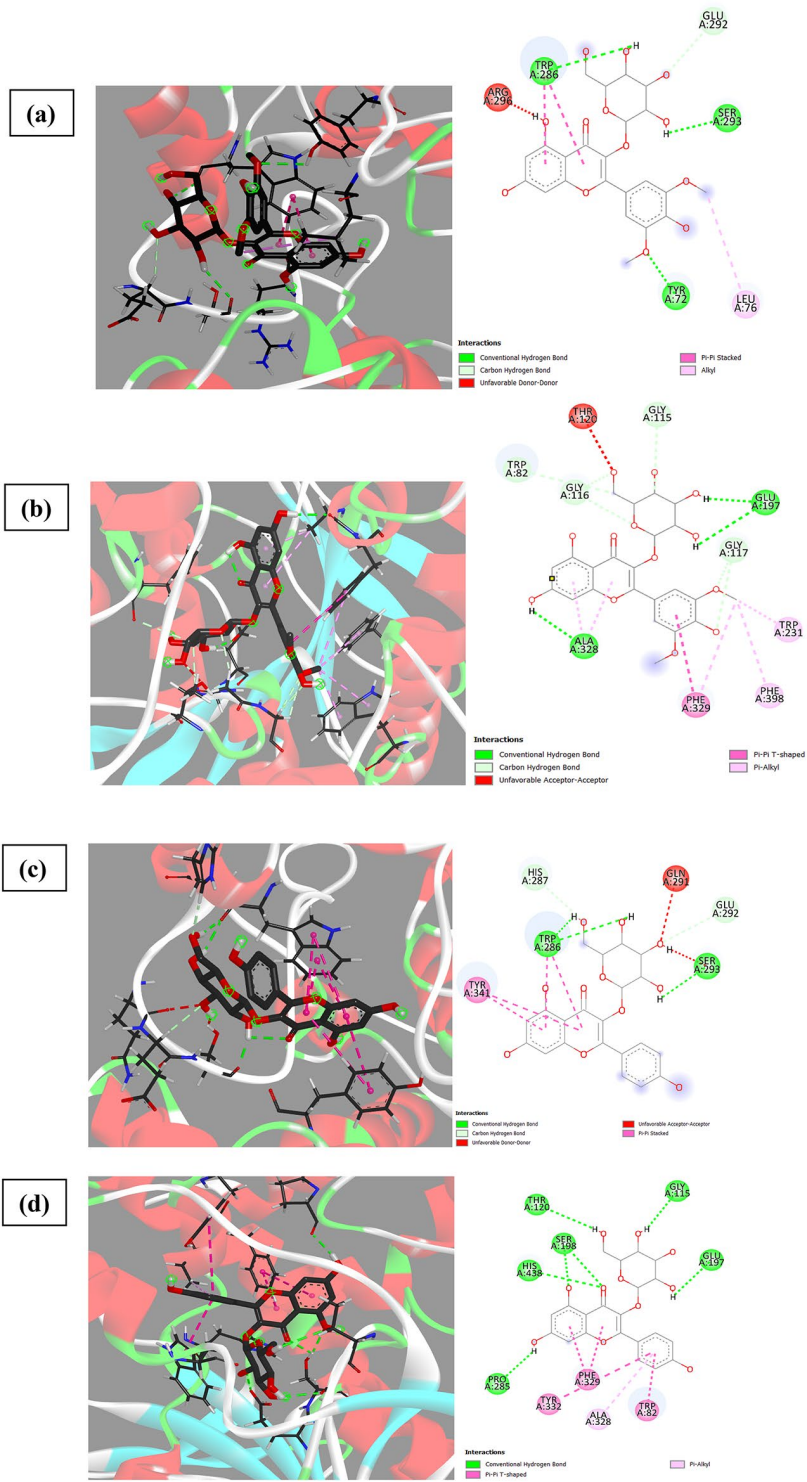

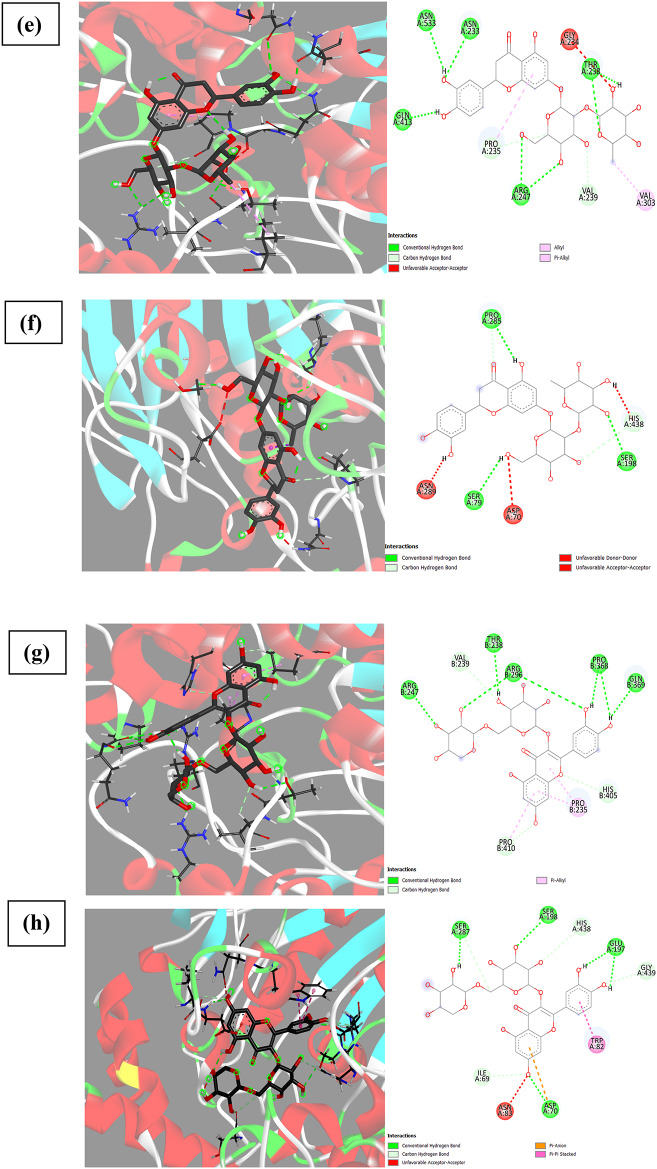
3D and 2D binding of the highly selective compounds to BChE compared to their binding to AChE, (**a**), (**b**) binding of syringetin-3-*O*-glucoside to AChE and BChE, respectively, (**c**), (**d**) binding of kaempferol-3-*O*-glucoside to AChE and BChE, respectively, (**e**), (**f**) binding of eriodictyol-7-*O*-neohesperidoside to AChE and BChE, respectively, (**g**), (**h**) binding of quercetin-3-*O*-arabinoglucoside to AChE and BChE, respectively.

The BChE active-site gorge has a volume that is ~ 200 Å 3 greater than the AChE gorge, allowing for the accommodation of compounds with varying orientations^45^. Therefore, docking of compounds inside BChE making several H- bonding with amino acids of the binding active site as Asp 70, Ser 79, 198, Trp 82, Gly 115,116,117, Thr 120, 284, Glu 197, Pro 285, Ala 328 and His 438. In addition, van der Waals forces (*Pi* or *Pi-Pi* t-shaped) with amino acids: Trp 82, 231, Ala 328, Phe 329, 398 and Try 332 (Fig. 12). Quercetin-3-*O*-arabinoglucoside and apigenin-6-*C*-glucoside − 7-*O*-glucoside achieved the highest binding affinity to BChE (− 10.8).

AChE’s active site is a narrow active-site gorge some 20Å deep and has two ligand binding sites: an acylation site (A-site) at the base of the gorge and a peripheral site (P-site) at its mouth. The P-site traps the substrate and can block the A-site, while the A-site catalyzes each substrate turnover. The P-site binds certain ligands and is spanned by residues W286 and D74^46,47^. Eriodictyol-7-*O*-neohesperidoside exhibited the highest affinity for AChE (−9.8), attributed to the hydrogen bonds formed with Asn 233, 533, Pro 235, Thr 238, Val 239, Arg 247, and Gln 413. Additionally, Val 303 contributed to a *pi*-interaction.

**Fig. 13:**
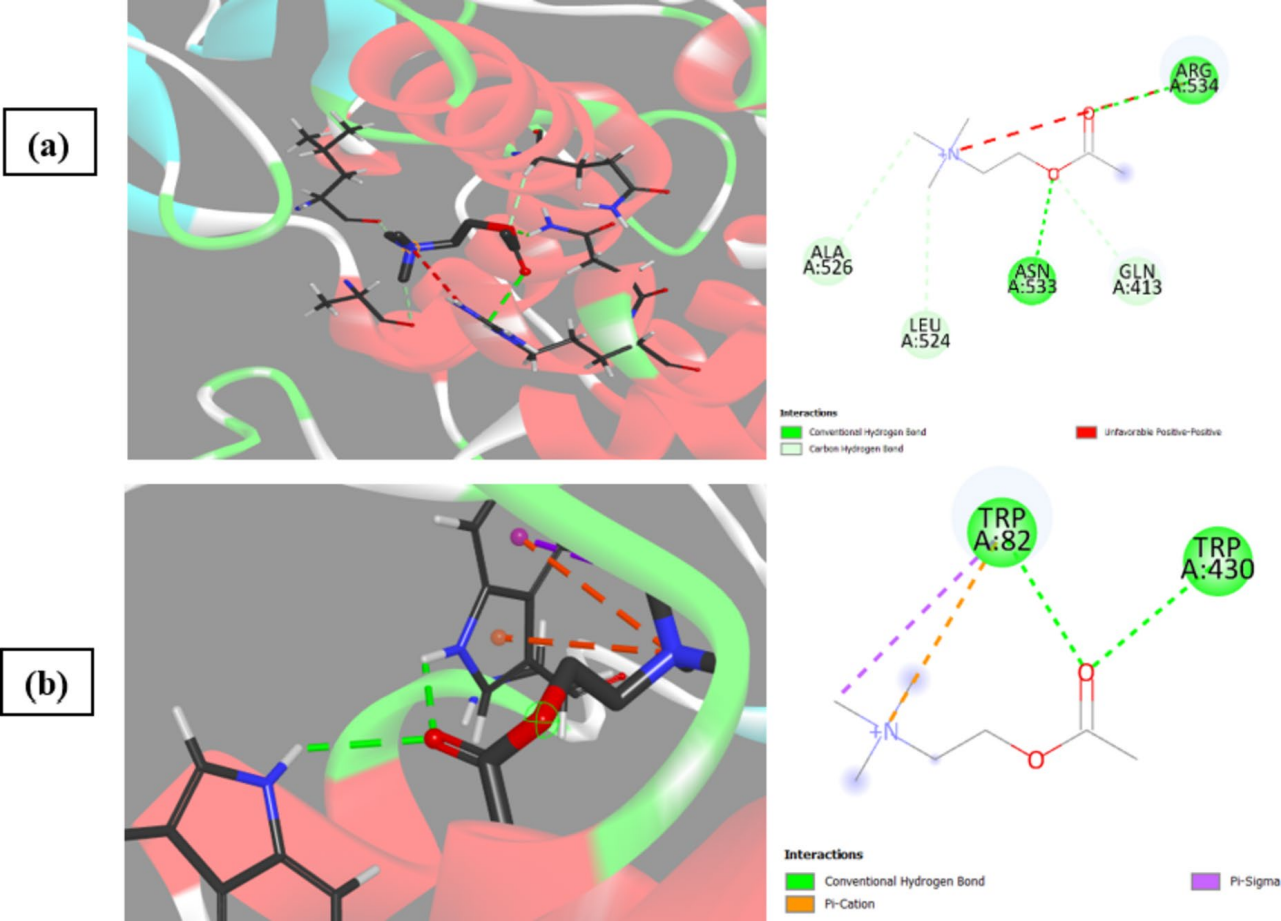
3D and 2D binding of acetylcholine as a ligand to (**a**) AChE and (**b**) BChE.

It has been observed that acetylcholine as a natural ligand has the same binding affinity for AChE and BChE (Fig. 13) while kaempferol-3-*O*-glucoside, isorhamnetin-3-*O*-rutinoside, delphinidin-3-*O*-(6’’-*O*-*α*-rhamnopyranosyl-*β*-glucopyranoside), quercetin-3-*O*-arabinoglucoside, and syringetin-3-*O*-glucoside exhibited the highest selectivity for BChE over AChE, with differences of 2.2, 2.0, 1.8, 1.7, and 1.6, respectively (Fig. 12). Based on our observations and conclusions, it can be stated that flavonoids achieve the highest affinity for either AChE or BChE due to the presence of 5-OH and 7-OH, increased substitution in Ring A, unsaturation, C-3 substitution of Ring C, and 3’,4’-OH of Ring B (more substitution increasing the affinity)^18^. The decrease in BChE inhibition may be attributed to the absence of carbonyl at C4(C = O) in Ring C, as in peonidine-3-*O*-glucoside and malvidin-3-galactoside, or no substitution at C3 or 3-OH, as in 3,3’,4’,5-tetrahydroxy-7-methoxyflavone, luteolin, luteolin-8-*C*-glucoside, gossypin, (±)-taxifolin, myricetin, quercetin, 3’-methoxy-4’,5,7-trihydroxyflavonol, diosmin, acacetin, and 4’,5,7-trihydroxyflavonol. The main difference in ligand affinity between BChE and AChE may be attributed to Ring B substitution^6–9,48^.

As observed in Fig. 12, the binding affinity for AChE was increased by changing the compounds from syringetin-3-*O*-glucoside/kaempferol-3-*O*-glucoside with a binding affinity of − 8.0/− 8.1 to eriodictyol-7-*O*-neohesperidoside/quercetin-3-*O*-arabinoglucoside with a binding affinity of −9.8/−9.1. This increase in binding affinity also led to an increase in the inhibition of BChE activity from − 10.0/−10.3 to −10.4/−10.8, respectively for the same compounds. The main reason for this observed increase is due to the bulk substitution of rings C and A at 3-*O*- and 7-*O*- positions, respectively.

## Conclusions

The results of our study provide evidence that *F. arabica* L. herb, which is widely used, may be effective in Alzheimer’s disease. This is due to its phenolic content, which has a significant inhibitory effect on the butyrylcholinesterase enzyme, as demonstrated by our in vitro analysis. Additionally, our findings suggest the possible modifications to the phenolic compounds which may increase their binding affinity or selectivity for BChE. These modifications were established through an in silico study and are thought to be promising for the development of newer and more effective anti-Alzheimer drugs.

## Electronic supplementary material

Below is the link to the electronic supplementary material.

Supplementary Material 1

Supplementary Material 2

## Data Availability

Data is provided within the manuscript and the supplementary information file.

## Declarations

### Declaration of AI use

We have not used AI-assisted technologies in creating this article.
