# Supplementary material for: UPLC-qTOF-MS/MS profiling of phenolic compounds in Fagonia arabica L. and evaluation of their cholinesterase inhibition potential through in-vitro and in-silico approaches
Source: Sci Rep. 2025 Feb 12;15:5244. doi: 10.1038/s41598-025-86227-0 (PMC11822067; doi:10.1038/s41598-025-86227-0)
Supplement: Supplementary file 1 — Supplementary Material 1 [file 41598_2025_86227_MOESM1_ESM.docx]

**Abbreviations**

| AChE | Acetylcholinesterase |
| --- | --- |
| AD | Alzheimer's disease |
| BChE | Butyrylcholinesterase |
| EtOAc | Ethyl acetate |
| *F. arabica* | *Fagonia arabica* L. |
| GA | Gallic acid |
| Glc | Glucose |
| IC_50_ | Half-maximal inhibitory concentration |
| *n*-BuOH | *n*-Butanol |
| MeCN  MeOH | Acetonitrile  Methanol |
| R | Rutin |
| RDA | Retro-Diels–Alder |
| ReSpect | Database for phytochemicals |
| Rha | Rhamnose |
| Rt | Retention time |
| SD | Standard Deviation |
| TIC | Total Ion Chromatogram |
| UPLC-qTOF-MS/MS | Ultra-Performance Liquid Chromatography-quadrupole Time-of-Flight- Mass Spectrometry |
