# Supplementary material for: UPLC-qTOF-MS/MS profiling of phenolic compounds in Fagonia arabica L. and evaluation of their cholinesterase inhibition potential through in-vitro and in-silico approaches
Source: Sci Rep. 2025 Feb 12;15:5244. doi: 10.1038/s41598-025-86227-0 (PMC11822067; doi:10.1038/s41598-025-86227-0)
Supplement: Supplementary file 2 — Supplementary Material 2 [file 41598_2025_86227_MOESM2_ESM.docx]

**Supplementary material**

**UPLC-qTOF-MS/MS profiling of phenolic compounds in *Fagonia arabica* L. and evaluation of their cholinesterase inhibition potential through *in-vitro* and *in-silico* approaches**

**Sarah A. Badawy^1^***, **Ahmed R. Hassan^1^, Marwa S. Abu Bakr^2^ & Abd El‑Salam I. Mohammed^3^**

^1^ Medicinal and Aromatic Plants Department, Desert Research Center, El-Matariya 11753, Cairo, Egypt

^2^ Department of Pharmacognosy, Faculty of Pharmacy (for Girls), Al-Azhar University, Nasr City 11651, Cairo, Egypt

^3^ Department of Pharmacognosy, Faculty of Pharmacy (for Boys), Al-Azhar University, Nasr City 13129, Cairo, Egypt

* Corresponding author

*E-mail address*: [sarahahmed.2252@azhar.edu.eg](mailto:sarahahmed.2252@azhar.edu.eg) (Sarah A. Badawy)

**Abstract**

*Fagonia arabica* L. is a widely used traditional medicinal herb. This study explored the flavonoid and phenolic acid content in the aerial parts of *F. arabica*, leading to the tentative identification of 42 compounds using Ultra-Performance Liquid Chromatography-Quadrupole Time-of-Flight Mass/Mass Spectrometry and analyzed with the phytochemical-focused RIKEN tandem mass spectral database (ReSpect) for identification based on authentic standards. The total phenolic and flavonoid content was measured in the ethyl acetate and butanol fractions. The flavonoid content in the ethyl acetate fraction was 101±1.43 µg Rutin/mg, compared to 6.48±0.29 µg rutin/mg in the butanol fraction. Similarly, the ethyl acetate fraction contained 199.14±1.58 µg gallic acid/mg, while the butanol fraction had 47.69±0.54 µg gallic acid/mg. Also, the study demonstrated the effectiveness of the different fractions of *Fagonia arabica* L. in inhibiting the butyrylcholinesterase enzyme, which is a key contributor to the progression of Alzheimer's disease. At a concentration of 0.45 mg/mL, the ethyl acetate fraction showed the highest efficiency, inhibiting butyrylcholinesterase by 50% (IC_50_). Based on the *in vitro* results, a molecular docking study suggested the selectivity of the tentatively identified compounds towards butyrylcholinesterase over acetylcholinesterase, as kaempferol-3-*O*-glucoside achieved the highest selectivity. This insight could inform potential modifications to enhance selectivity, which may be applied in the synthesis, semi-synthesis, and development of novel treatments for Alzheimer's disease.

**Keywords:** *Fagonia arabica*; UPLC-qTOF-MS/MS; Flavonoids; Butyrylcholinesterase inhibitors; Acetylcholinesterase inhibitors; Docking study.

1. ***In-silico* study:**

**Table S1:** Structures, binding energies and selectivity of the phenolic constituents of *F. arabica* to Cholinesterases

| **Compound No.** | **Molecule** | **Docking score**  **AChE *Cal/mol*** | **Docking score**  **BChE *Cal/mol*** | **Difference in scores**  **[BChE-AChE]** |
| --- | --- | --- | --- | --- |
| **1** | Kaempferol-3-*O*-glucoside   | -8.1 | -10.3 | 2.2 |
| **2** | Isorhamnetin-3-*O*-rutinoside   | -8.1 | -10.1 | 2.0 |
| **3** | Delphinidin-3*-O*-(6''-*O*-*α*-rhamnopyranosyl-*β-*glucopyranoside)   | -8.8 | -10.6 | 1.8 |
| **4** | Quercetin-3-*O*-arabinoglucoside   | -9.1 | -10.8 | 1.7 |
| **5** | Syringetin-3-*O*-glucoside   | -7.8 | -9.4 | 1.6 |
| **6** | Kaempferol-3-*O-α*-L-arabinoside   | -8.2 | -9.6 | 1.4 |
| **7** | 3', 4', 5, 7-tetrahydroxyflavanone   | -8.0 | -9.4 | 1.4 |
| **8** | Apigenin-6-*C*-glucoside -7-*O*-glucoside   | -9.5 | -10.8 | 1.3 |
| **9** | Apigenin 8-*C*-glucoside   | -9.6 | -10.7 | 1.1 |
| **10** | Kaempferol-3-*O*-(6-*p*-coumaroyl)-glucoside   | -9.6 | -10.7 | 1.1 |
| **11** | 3, 3', 4', 5-tetrahydroxy-7-methoxyflavone   | -8.0 | -9.1 | 1.1 |
| **12** | Luteolin   | -7.9 | -9.0 | 1.1 |
| **13** | Gossypin   | -8.3 | -9.3 | 1.0 |
| **14** | (±)-Taxifolin   | -8.4 | -9.3 | 0.9 |
| **15** | Luteolin-8-*C*-glucoside   | -8.5 | -9.3 | 0.8 |
| **16** | Rosmarinic acid   | -7.8 | -8.6 | 0.8 |
| **17** | Myricetin   | -8.4 | -9.1 | 0.7 |
| **18** | kaempferol-3-*O*-robinoside-7-*O*-rhamnoside   | -9.5 | -10.1 | 0.6 |
| **19** | Eriodictyol-7-*O*-neohesperidoside   | -9.8 | -10.4 | 0.6 |
| **19** | Quercetin-3,4'-*O*-di-*β*-glucopyranoside   | -8.8 | -9.3 | 0.5 |
| **20** | Quercetin   | -8.2 | -8.7 | 0.5 |
| **21** | Petunidin-3-*O*-*β*-glucopyranoside   | -8.8 | -9.2 | 0.4 |
| **22** | Okanin-4'-*O*-glucoside   | -9.0 | -9.3 | 0.3 |
| **23** | Diosmin   | -9.6 | -9.7 | 0.1 |
| **24** | Malvidin-3-*O*-glucoside   | -8.9 | -9.0 | 0.1 |
| **25** | Naringenin   | -8.9 | -9.0 | 0.1 |
| **26** | 3, 5, 7-trihydroxy-4'-methoxyflavone   | -8.8 | -8.6 | ____ |
| **27** | Peonidin-3,5-*O*-di-*β*-glucopyranoside   | -9.5 | -9.3 | ____ |
| **28** | 3'-methoxy-4',5,7-trihydroxyflavonol   | -9.0 | -8.6 | ____ |
| **29** | 1-*O-β*-D-glucopyranosyl sinapate   | -7.8 | -7.2 | ____ |
| **30** | Peonidine-3-*O*-glucoside   | -9.0 | -8.9 | ____ |
| **31** | Malvidin-3-galactoside   | -8.9 | -8.8 | ____ |
| **32** | Resveratrol   | -8.0 | -7.7 | ____ |
| **33** | Acacetin   | -8.7 | -8.5 | ____ |
| **34** | 4',5,7-trihydroxyflavonol   | -9.0 | -8.6 | ____ |
| **35** | Maritimetin-6-*O*-glucoside   | -9.5 | -9.4 | ____ |
| **36** | 3-(4-hydroxy-3,5-dimethoxyphenyl)-2-propenoic acid   | -7.0 | -6.6 | ____ |
